# Integrated mRNA and Small RNA Sequencing Reveals microRNAs Associated With Xylem Development in *Dalbergia odorifera*

**DOI:** 10.3389/fgene.2022.883422

**Published:** 2022-04-25

**Authors:** Wenxiu Zhao, Xiangxu Meng, Jiahong Xu, Zijia Liu, Yangyang Hu, Bingyu Li, Jinhui Chen, Bing Cao

**Affiliations:** ^1^ Key Laboratory of Genetics and Germplasm Innovation of Tropical Special Forest Trees and Ornamental Plants, Ministry of Education/Engineering Research Center of Rare and Precious Tree Species in Hainan Province, School of Forestry, Hainan University, Haikou, China; ^2^ Sanya Nanfan Research Institute of Hainan University, Hainan Yazhou Bay Seed Laboratory, Sanya, China

**Keywords:** *Dalbergia odorifera*, microRNA (miRNA), xylem differentiation, RNA sequencing, species-specific noncoding RNAs

## Abstract

*Dalbergia odorifera* is a rare and precious rosewood specie, whose wood is a very high-quality material for valuable furniture and carving crafts. However, limited information is available about the process of wood formation in *D. odorifera*. To determine genes that might be closely associated with the xylem differentiation process, we analyzed the differentially expressed genes (DEGs) and microRNAs (miRNAs) from specific xylem tissues of *D. odorifera* by RNA sequencing (RNA-seq) and small RNA sequencing (small RNA-seq). In total, we obtained 134,221,955 clean reads from RNA-seq and 90,940,761 clean reads from small RNA-seq. By comparing the transition zone (Dotz) and sapwood (Dosw) samples, a total of 395 DEGs were identified. Further analysis revealed that DEGs encoded for WRKY transcription factors (eight genes), lignin synthesis (*PER47*, *COMT*, *CCR2*), cell wall composition (*UXS2*), gibberellin synthesis (*KAO2*, *GA20OX1*), jasmonic acid synthesis (*OPR2, CYP74A*), and synthesis of flavonoids (*PAL2*) and terpenoids (*CYP71A1*). Subsequently, a preliminary analysis by small RNA-seq showed that the expressions of 14 miRNAs (such as miR168a-5p, miR167f-5p, miR167h-5p, miR167e, miR390a, miR156g, novel_52, and novel_9) were significantly different between Dotz and Dosw. Further analysis revealed that the target genes of these differentially expressed miRNAs were enriched in the GO terms “amino acid binding,” “cellulase activity,” and “DNA beta-glucosyltransferase activity”. Further, KEGG pathway annotation showed significant enrichment in “fatty acid elongation” and “biosynthesis of unsaturated fatty acids”. These processes might be participating in the xylem differentiation of *D. odorifera*. Next, expression correlation analysis showed that nine differentially expressed miRNAs were significantly negatively associated with 21 target genes, which encoded for proteins such as *pyrH*, *SPL6*, *SPL12*, *GCS1*, and *ARF8*. Overall, this is the first study on miRNAs and their potential functions in the xylem development of *D. odorifera*, which provides a stepping stone for a detailed functional investigation of *D. odorifera* miRNAs.

## Introduction

*Dalbergia odorifera* is a semi-deciduous tree that belongs to the Leguminosae family (Vatanparast et al., 2013; Wariss et al., 2017), with the characteristics of easy germination, good tolerance to drought and barren soil, high disease resistance, and high adaptability (Liu et al., 2017; Hong et al., 2020). The heartwood of *D. odorifera* is exquisite in color and pattern, hard in texture, and has a unique aroma. Therefore, it has always been considered as a very high-quality material for making precious furniture, musical instruments, decorations, and handicrafts (Meng et al., 2019). Due to its extremely high application value, *D. odorifera* was over-exploited, the wild resources were extensively destroyed. The natural forests became increasingly scarce and were almost extinct (Liu et al., 2019). The slow speed of heartwood formation further prevents the existing resources of *D. odorifera* from meeting the demand of domestic and foreign wood markets (Cui et al., 2020). Therefore, it is urgent to cultivate *D. odorifera* trees with good wood quality.

Wood formation involves the biosynthesis and deposition of lignin and cellulose on the cell wall, which comprises a complex network of multiple genes (Mizrachi and Myburg, 2016; Wang Y.-H. et al., 2018). Further, the heartwood formation also involves the generation of special metabolites during its transformation from sapwood (Ye and Zhong, 2015; Celedon and Bohlmann, 2018). The main components of heartwood in *D. odorifera* are essential oils and flavonoids (The, 2017; Zhao et al., 2020). Essential oils are secondary metabolites having aromatic odor and volatile properties, and include terpenoids, aromatic compounds, aliphatic compounds, and compounds containing sulfur and nitrogen (Yang et al., 2022). The biosynthesis and accumulation of lignin, cellulose, essential oils, and flavonoids are important factors that influence xylem differentiation and wood formation in *D. odorifera*. Still, the identity of genes and other regulators involved in the process of xylem differentiation of *D. odorifera* under natural growth remain poorly known.

Wood refers to all tissues within the vascular cambium in the hard stems of perineal plants. Usually, in the cross-section of the wood, we can observe the heartwood is the dark and hard textured central area, while the sapwood is the light and soft textured outer area (Islam et al., 2012; Mizrachi and Myburg, 2016; Chen et al., 2018). As the trees grow, sapwood gradually transforms into the heartwood. This process usually takes place in a narrow transition zone, which comprises living cells that consume reserves such as starch (Bergström, 2003). In general, the transition zone is one to two rings wide and is adjacent to the heartwood (Celedon and Bohlmann, 2018). The xylem tissue of the transition zone is often regarded as an important material for studying wood formation. For example, in *Taiwania cryptomerioides*, the molecular mechanism of the autolysis of the cellular components of ray parenchyma cells in the transition zone, during the heartwood formation, was elucidated by transcriptome sequencing of sapwood and transition zone, in combination with the microscopy and high-performance liquid chromatography technology (Yeh et al., 2020). In *Pinus sylvestris*, transcriptomic sequencing revealed that stilbene and resinic acid were synthesized in transition zone and sapwood, respectively (Lim et al., 2016). These studies suggest that sapwood and transition zone are ideal plant parts for studying the dynamics of gene expression and molecular regulatory mechanisms of wood formation (Yeh et al., 2020).

MicroRNAs (miRNAs) now form an important component of machinery that regulate gene expression. MiRNAs are a class of small, single-strand non-coding RNAs that are approximately 21 nucleotides in length (Rogers and Chen, 2013; Yu et al., 2017). They exert a negative regulation on the expression of target genes through sequence-based complementarity, resulting in mRNA cleavage or translation repression (Chen et al., 2015; Song et al., 2019; Wang M et al., 2021). Studies have shown that miRNAs play important roles in plant growth and development, including organ morphogenesis, hormone secretion, and stress response (Aukerman and Sakai, 2003; Sunkar et al., 2012; Dong et al., 2022). For example, in *Osmanthus fragrans*, miR858 affects flavonoid content in flower tissues by negatively regulating the *MYB1* gene (Shi et al., 2021). Further, some miRNAs, such as miR397a (Lu et al., 2013), miR875 (Zhao et al., 2015), miR257 (Chen et al., 2016), and miR475b (Xiao et al., 2017), have been identified to participate in different processes of wood formation. However, the expression profile and functions of miRNAs in *D. odorifera* have not been reported. A detailed understanding of the complex molecular mechanism of *D. odorifera* xylem differentiation has also been lacking.

Here, we have investigated the expression profile of miRNAs and their potential role in regulating xylem differentiation in *D. odorifera*. We performed small RNA sequencing (small RNA-seq) and mRNA sequencing (mRNA-seq) analyses from three biological replicates of two tissue types (sapwood and transition zone) of *D. odorifera*. To the best of our knowledge, this is the first study to identify miRNAs of *D. odorifera* and describe their potential role in xylem differentiation. This study will broaden our understanding of the complex molecular mechanism of *D. odorifera* xylem differentiation. The results of sequencing analysis provide abundant candidate miRNAs and mRNAs, which are important for the innovation of *D. odorifera* germplasm resources and the cultivation of good wood quality varieties.

## Materials and Methods

### Plant Materials

The three well-developed *D. odorifera* trees (about the age of 7 years) were grown at an artificial nursery of Hainan Province in China (19°38′56″N, 110°14′29″E). These three individual trees, which have formed heartwood, were selected as biological replicates without any treatment. These three trees were used for all the experiments. Samples of sapwood (Dosw1, Dosw2, Dosw3) and transition zone (Dotz1, Dotz2, Dotz3) were taken from xylem tissues near the cambium and near the heartwood of each tree, respectively, by following the previously described protocols (Yeh et al., 2020). All the samples were isolated from the trees with the help of a sharp chisel after removing the bark, and the tissues were immediately frozen in liquid nitrogen and stored at −80°C until RNA isolation.

### Library Construction and Sequencing

Total RNA from Dotz and Dosw groups of samples was extracted by the CTAB method. Agarose gel electrophoresis (2% agarose gel) was used to evaluate RNA degradation and contamination, and a micro-spectrophotometer was used to assess the purity of RNA (260/280 ratio: 1.9–2.2, 260/230 ratio: ≥ 2.0). RNA integrity was evaluated with the help of RNA Nano 6000 Assay Kit of the Agilent Bioanalyzer 2,100 system (Agilent Technologies, CA, United States), and samples with an RNA integrity number (RIN) greater than 7.0 were used for further experiments.

Magnetic beads with oligo (dT) were used to enrich mRNA by binding to its polyadenylated tail through A-T complementary pairing. A fragmentation buffer was used for fragmenting the mRNA. cDNA was synthesized using random hexamer primers, buffer, dNTPs, and DNA polymerase I. Double-stranded cDNA was purified by AMPure XP beads, and subjected to end repair, the addition of the poly-A tail, ligation of the sequencing linker, and fragment size selection. Finally, the cDNA libraries were subjected to PCR enrichment and sequenced with the Illumina HiSeq 2,500. After the original data was filtered, the redundancy was removed to obtain clean reads. The HISAT2 software was used to compare the clean reads of RNA-Seq to the reference genome sequence of *D. odorifera*.

The NEBNext^®^ Multiplex small RNA library prep set was used to generate small RNA-seq libraries for Illumina^®^ (NEB, Ipswich, MA, United States.) by following the manufacturer’s instructions. Then, the TruSeq SR Cluster Kit v3-cBot-HS (Illumina) was used to generate a cluster on cBot cluster generation system. Finally, all small RNA-seq libraries were sequenced on an Illumina HiSeq 2,500 platform.

### Analysis of miRNA and mRNA Expression Profiles

After sequencing, clean reads were obtained by removing the reads containing poly-N, ploy A/T/G/C, with 5′ adapter contaminants, without 3′adapter or the insert tag, and the low-quality reads. In addition, Q20, Q30, and GC-content of the raw reads also were calculated. At the end, all the downstream analyses were performed on sequences ranging of 18–30 bp in length. Bowtie was used to locate/align the small RNAs to the reference genome of *D. odorifera* and analyze the distribution of small RNAs over the reference genome. The reads that mapped onto the reference genome of *D. odorifera* were compared with sequences in miRBase (20.0) to obtain the known miRNA; whereas novel miRNAs were predicted with the help of miREvo (Wen et al., 2012) and mirDeep2 (Friedlander et al., 2012). The expression level of miRNA was estimated by the TPM (transcript per million) value. The DESeq R package (3.0.3) was used to perform differential expression analysis of Dotz and Dosw with the |log_2_ (fold change)| ≥ 1 and *p*-value < 0.05 as the threshold.

Clean reads of RNA-seq were obtained by removing low-quality sequencing fragments from the raw reads. Then, the read count of each gene was obtained by mapping the clean reads to the reference genome of *D. odorifera* with the help of HISAT2. The expression levels of mRNAs were estimated by the calculating FPKM (fragments per kilobase of exon model per million) values. A |log_2_ (fold change)| ≥ 1 and q-value < 0.05 were used as criteria to screen differentially expressed genes (DEGs) between Dotz and Dosw samples.

### Prediction of the Potential Target Genes of miRNA

The psRobot is focused on plant small RNA analysis, which has been widely used for target gene predictions (Wu et al., 2012; Zhao et al., 2020; Liao et al., 2021). Therefore, we used its online version (http://omicslab.genetics.ac.cn/psRobot/target_prediction_1.php/) to predict the potential target genes of miRNAs, using the default parameters (Wu et al., 2012). Then, correlation analysis of accumulation of target genes and miRNAs was performed. When the expression levels of target genes had a strong negative correlation of at least −0.8 (*p* < 0.05) with miRNAs, they were selected for further analysis. All DEGs and the target genes of miRNAs were mapped to individual terms in the GO database (http://www.geneontology.org/) and the number of genes per term was calculated. Then, the GOseq software was used for GO enrichment analysis of DEGs. Analysis of gene regulatory pathways in the KEGG pathway database (http://www.genome.jp/kegg/pathway.html/) was performed with the help of KOBAS (3.0) software. Finally, Cytoscape 3.9.0 was used to construct a co-expression network graph.

### Validation of miRNA and Gene Expression by RT-qPCR

cDNAs were synthesized by reverse transcription of total RNA from six *D. odorifera* samples (Dosw1, Dosw2, Dosw3, Dotz1, Dotz2, and Dotz3). Gene-specific primers for the targets were designed with the help of Primer Premier v5 software (Supplementary Table S1). Four important DEGs (*WRKY22*, *AP2*, *FPP7*, and *PAL2*), five miRNA target genes (*SPL12*, *ARF8*, *FH20*, *GCS1*, and *MMT1*) of miRNA-mRNA correlation network, and six differentially expressed miRNAs (miR156g, miR167e, miR168a-5p, novel_9, novel_15, and novel_52) were chosen to verify expression levels. For the genes, RT-qPCR analysis was conducted with TB Green^®^ Premix Ex Taq™ (Tli RNaseH Plus; Takara, Beijing, China) following the manufacturer’s recommendations. The amplification was performed on a preheated (94°C) thermal cycler, and samples were incubated at 94°C for 2 min, followed by 40 cycles of 95 °C for 5 s and 60°C for 30 s. The *actin* gene served as an internal control for normalization (Meng et al., 2019). We used the miRNA RT-qPCR Detection Kit (Aidlab, Beijing, China) for RT-qPCR analysis of miRNAs, following the manufacturer’s recommendations. PCR amplification was performed at a preheated (94°C) thermal cycler and samples were incubated at 94 °C for 2 min, followed by 40 cycles of 94°C for 15 s and 60°C for 40 s. The *U6* gene served as an internal control for normalization (Meng et al., 2022). The 2^–△△Ct^ method was used to calculate the expression levels of the miRNAs and genes against the internal controls (Schmittgen and Livak, 2008). Three technical replicates per sample were analyzed to ensure reproducibility and reliability.

## Results

### mRNA Expression Profile of Xylem Differentiation in *D. odorifera*

**FIGURE 1:**
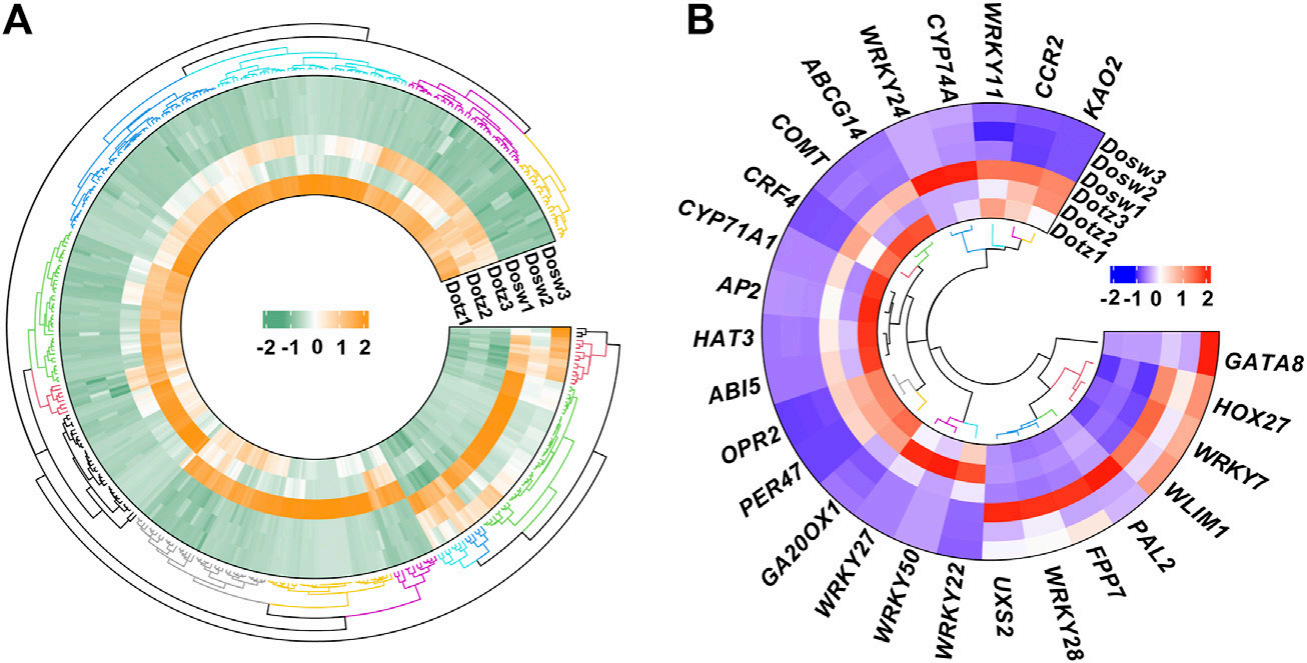
Expression profiles of mRNAs in *D. odorifera*. Hierarchical clustering of all differentially expressed mRNAs **(A)**. Hierarchical clustering of expression of important differentially expressed genes **(B)**.

To understand the molecular mechanism of xylem differentiation, six cDNA libraries from the Dotz and Dosw of *D. odorifera* were sequenced. RNA-seq generated 20,567,345 (Dotz1), 20,373,919 (Dotz2), 25,875,484 (Dotz3), 21,492,446 (Dosw1), 22,895,259 (Dosw2) and 23,017,502 (Dosw3) clean reads (Table 1). Of these, 19,643,871 (Dotz1), 19,430,607 (Dotz2), 24,690,387 (Dotz3), 20,041,706 (Dosw1), 21,867,262 (Dosw2) and 21,684,789 (Dosw3) clean reads, respectively, were mapped to the *D. odorifera* genome with mapping ratios of 93.25–95.51% (Table 1). These results show that RNA-seq captured a significant portion of the genes in the genome of *D. ororifera*. For differential analysis of gene expression between the Dotz and Dosw groups, the FPKM values were used to normalize the reads from RNA-seq, and a cutoff of |log_2_ (fold change)| ≥ 1 and q-value <0.05 was used. A total of 395 mRNAs were differentially accumulated, of which 72 were up- and 323 were down-regulated, respectively, in Dotz compared to Dosw (Figure 1A; Supplementary Table S2). All 395 differentially expressed mRNAs (Supplementary Table S2) obtained from Dotz and Dosw were used for subsequent analysis.

**TABLE 1:** Summary of mRNA sequencing datasets.

Sample	Raw reads	Clean reads	Q20 (%)	Q30 (%)	GC (%)	Mapping rate (%)
Dotz1	21,748,426	20,567,345	96.42	90.26	45.48	93.25
Dotz2	21,250,794	20,373,919	96.51	90.47	45.19	95.51
Dotz3	27,412,017	25,875,484	97.17	92.16	45.41	94.21
Dosw1	22,942,939	21,492,446	96.40	90.41	46.17	95.51
Dosw2	24,101,319	22,895,259	96.80	91.18	45.46	95.37
Dosw3	23,998,691	23,017,502	96.80	91.16	45.70	95.42

### Functional Annotation of the DEGs

**FIGURE 2:**
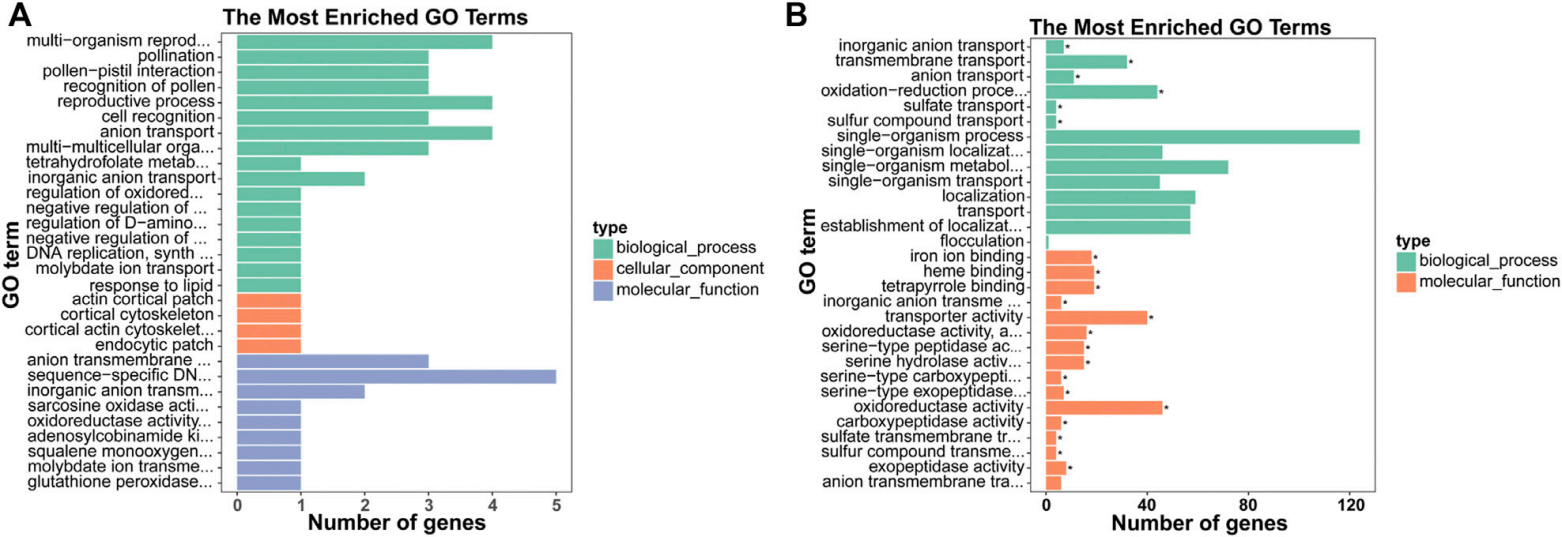
GO analysis of the biological functions of mRNAs. The x-axis represents the number of genes, and the y-axis represents the GO term. GO enrichment analysis of the up- **(A)** and down-regulated **(B)** mRNAs are presented.

**FIGURE 3:**
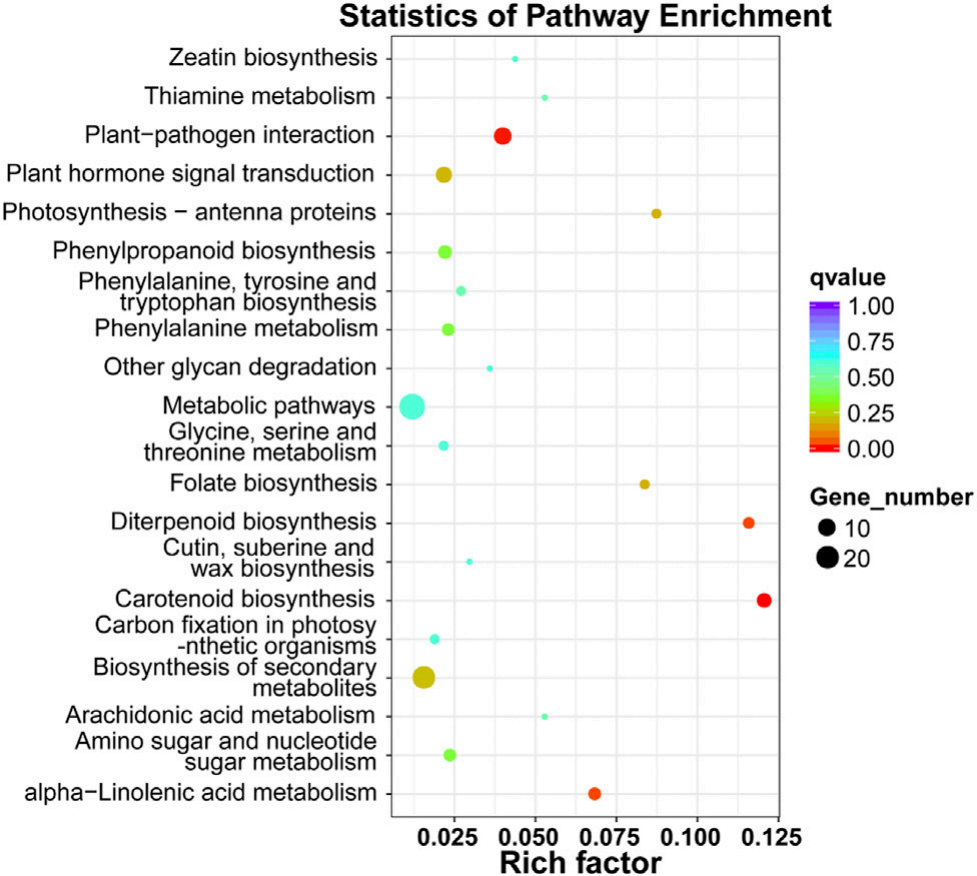
KEGG pathway enrichment analysis of differentially expressed mRNAs. The x-axis represents the rich factor, whereas the y-axis represents the KEGG pathway term. The size of the dots represents the number of genes, and the color of the dots represents the qvalue.

To annotate and reveal the function of DEGs in different tissues of xylem, we used GO classification. Up-regulated mRNAs were associated with 11 molecular functions, 49 biological processes, and four cellular components (*p*-value < 0.05), including “anion transport,” “anion transmembrane transporter activity,” and “inorganic anion transport” associated with ion transport (Figure 2A). Down-regulated mRNAs were associated with 41 molecular functions, 54 biological processes, and three cellular components (*p*-value < 0.05), including “inorganic anion transmembrane transporter activity”, “transmembrane transport”, “sulfate transport” and “sulfate transmembrane transporter activity” terms associated with anion transport (Figure 2B). The DEGs were further referenced through the KEGG database. Eight KEGG pathways (*p*-value < 0.05) were significantly enriched, including “carotenoid biosynthesis,” “diterpenoid biosynthesis,” and “alpha-linolenic acid metabolism” (Figure 3).

Functional annotation of DEGs revealed the processes, such as the transcriptional regulation, lignin synthesis, flavonoids, and terpenoids synthesis, which could play important roles in the xylem differentiation of *D. odorifera*. We found that 11 DEGs might encode for transcription factors, of which eight belong to the WRKY transcription factor family (Figure 1B). Four genes (*PER47* (Peroxidase 47), *COMT* (Caffeic acid 3-O-methyltransferase), *CCR2* (Cinnamoyl-CoA reductase 2), and *UXS2* (UDP-glucuronic acid decarboxylase 2)) are related to lignin synthesis and cell wall composition, two genes (*KAO2* (Ent-kaurenoic acid oxidase 2) and *GA20OX1* (Gibberellin 20 oxidase 1)) are part of gibberellin synthesis, two genes (*OPR2* (12-oxophytodienoate reductase 2) and *CYP74A* (Allene oxide synthase)) participate in jasmonic acid synthesis, and two genes (*PAL2* (Phenylalanine ammonia-lyase 2) and *CYP71A1* (Cytochrome P450 71A1)) are related to flavonoids and terpenoids synthesis (Figure 1B).

### Sequencing of Small RNAs

Small RNA-seq was performed to unveil the possible role of miRNAs in regulating gene expression during xylem differentiation in *D. odorifera*. 15,809,909 (Dotz1), 15,412,631 (Dotz2), 15,469,161 (Dotz3), 15,422,891 (Dosw1), 15,394,233 (Dosw2) and 15,755,203 (Dosw3) raw reads were obtained from the small RNA libraries generated from the Dotz and Dosw groups. After removing connectors and low-quality reads, a total of 45,496,409 (Dotz) and 45,444,352 (Dosw) clean reads were obtained (Table 2). The length of most clean reads ranged between 21 and 24 nucleotides (Supplementary Figure S1). In comparison by Bowtie software, 90.87–95.30% of small RNA reads matched with the genome sequence of *D. odorifera* (Table 2).

**TABLE 2:** Summary of small RNA sequencing datasets.

Sample	Raw reads	Clean reads	Q20 (%)	Q30 (%)	GC (%)	Mapping rate (%)
Dotz1	15,809,909	15,359,940	97.42	92.25	50.44	94.25
Dotz2	15,412,631	15,004,955	97.17	91.47	50.13	90.87
Dotz3	15,469,161	15,131,514	97.43	92.22	50.08	93.12
Dosw1	15,422,891	15,024,534	97.33	91.99	50.81	95.30
Dosw2	15,394,233	15,027,909	97.35	91.90	50.16	93.03
Dosw3	15,755,203	15,391,909	97.36	91.88	49.18	95.20

### Identification of Known and Novel miRNA

**FIGURE 4:**
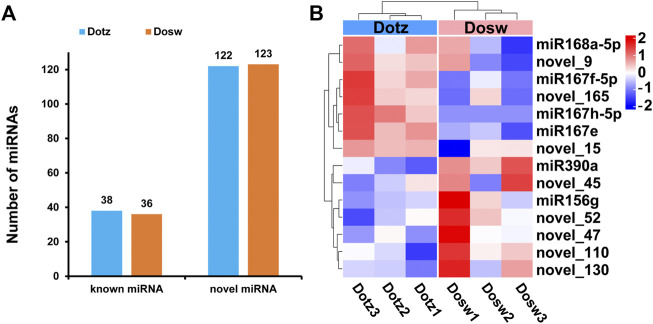
The expression profile of miRNAs in xylem of *D. odorifera*. **(A)** The number of known and novel miRNAs. The x-axis represents the type of miRNAs, and the y-axis represents the number of miRNAs. **(B)** Hierarchical clustering of all differentially expressed miRNAs.

The 18–30 nt clean reads that mapped to the reference genome were compared to sequences in miRBase to identify conserved/known miRNAs. Subsequently, novel miRNAs were predicted using miREvo and miRDeep2 tools on the basis of structural characteristics of miRNA. We identified 38 known miRNAs in Dotz and 36 in Dosw samples, respectively. Together, these accounted for a total of 40 known miRNAs that belonged to 22 miRNA-families (Figure 4A; Supplementary Table S3; Supplementary Figure S2). Further analysis of the distribution of the first base of these known miRNAs showed that “U” was the most dominant base (Supplementary Table S4; Supplementary Figure S3). Also, a total of 123 novel miRNAs were identified, where 122 were present in Dotz and all 123 in Dosw (Figure 4A; Supplementary Table S5; Supplementary Figure S4). The statistical analysis of the distribution of the first base of these novel miRNAs also found that “U” was the most dominant base (Supplementary Table S6; Supplementary Figure S5).

### Differentially Expressed miRNAs and Their Targets

To understand the miRNA-driven mechanism of regulation of xylem differentiation, miRNAs that accumulated differentially between the two tissue types were identified by the TPM method. Compared with Dosw, four known (miR168a-5p, miR167f-5p, miR167h-5p, and miR167e) and three novel miRNAs (novel_15, novel_9, and novel_165) were up-regulated in Dotz, whereas two known (miR390a, miR156g) and five novel miRNAs (novel_45, novel_52, novel_110, novel_47, and novel_130) were down-regulated (Figure 4B). Among these differentially expressed miRNAs, the most up-regulated miRNAs were miR167e (52.21 fold), miR167h-5p (20.53 fold), and miR167f-5p (16.33 fold). The top down-regulated miRNAs were miR390a (52.75 fold), novel_45 (21.44 fold), and novel_52 (17.17 fold) (Supplementary Table S7).

To understand the potential function of differentially expressed miRNAs, 1,056 candidate target genes were predicted with the help of psRobot. Then, the target genes with a strong negative correlation of at least −0.8 (*p* < 0.05) with miRNAs were selected for further analysis (Supplementary Table S8). We, thus obtained 21 putative target genes for nine differentially expressed miRNAs. Subsequently, GO enrichment analysis indicated that these 21 target genes are associated with 42 GO terms (*p*-value < 0.05) from biological processes, molecular functions, and cellular components. Few examples of such processes included “amino acid binding,” “cellulase activity,” and “DNA beta-glucosyltransferase activity” (Supplementary Figure S6A). Further, KEGG enrichment indicated that the target genes were enriched in five pathways (*p*-value < 0.05), including “protein processing in endoplasmic reticulum,” “fatty acid elongation,” and “biosynthesis of unsaturated fatty acids” (Supplementary Figure S6B).

### A Key miRNA-mRNA Regulatory Network During Xylem Differentiation of *D. odorifera*

**FIGURE 5:**
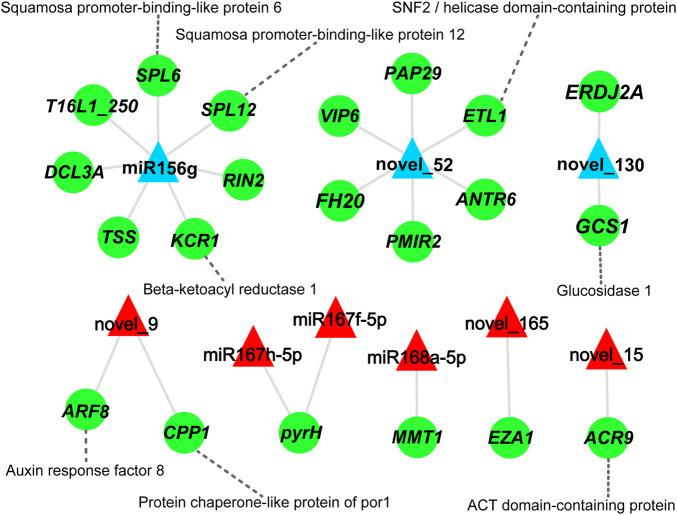
miRNA-mRNA correlation network. Green circles represent the genes, red and blue triangles represent up- and down-regulated miRNAs, respectively. The gray solid line represents the targeted regulatory relationship between miRNAs and genes, and the gray dotted line shows the protein encoded by the gene.

To explore the relationship between miRNAs and mRNAs during xylem differentiation of *D. odorifera*, a regulatory network diagram of the nine differential expression miRNAs and their 21 target genes was constructed (Figure 5; Supplementary Table S8). It was evident that one miRNA could target 1-7 mRNAs, among which miR156g and novel_52 could negatively regulate seven and six mRNAs, respectively, while miR167h-5p and miR167f-5p could simultaneously regulate one mRNA (*pyrH*, Uridylate kinase). Similarly, miR156g might target the transcripts of Squamosa promoter-binding-like protein 6 (*SPL6*), which is involved in programmed cell death (Wang Q.-L. et al., 2018), as well as the *SPL12*. A novel miRNA, novel_130, might target mRNA of mannosyl-oligosaccharide glucosidase (*GCS1*), involved in cellulose synthesis. The up-regulated miRNA novel_9 was predicted to target auxin response factor 8 (*ARF8*) that is involved in auxin dynamics balance (Tian et al., 2004) (Figure 5).

### Validation of Gene Expression Levels by RT-qPCR

**FIGURE 6:**
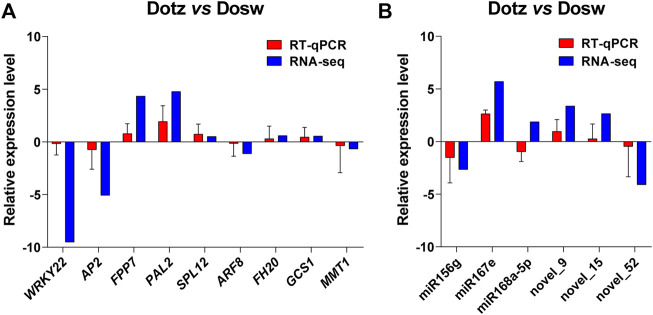
RT-qPCR of the expression levels of miRNAs and genes in Dosw and Dotz from the xylem in *D. odorifera*. The accumulation levels of mRNAs of the genes **(A)**, and miRNAs **(B)** in RT-qPCR and RNA-seq analyses are presented. The *actin* was used as an internal control for genes, and the *U6* was used as the internal control for miRNAs.

We determined the relative transcript levels of miRNAs and genes identified from DEGs and the interaction network by RT-qPCR, and observed that only miR168a-5p was different with RNA-seq. The expression patterns of all nine genes and other miRNAs estimated by RT-qPCR and RNA-seq followed a similar trend in Dotz and Dosw samples. Although the fold-change (FC) values calculated by sequencing did not exactly match the expression values obtained by RT-qPCR, the expression profiles were basically consistent for all tested miRNAs and genes (Figure 6). These analyses validate the reliability of the gene expression values generated from sequencing results.

## Discussion

*D. odorifera* is an important, rare, and precious rosewood. However, only a limited number of studies (Cui et al., 2019) are available that describe the molecular mechanism of wood formation of *D. odorifera*, which is comprised of multiple and complex pathways. RNA-seq technology now provides a convenient tool for a better understanding of the mechanisms of plant growth and development at the molecular level (Yeh et al., 2020). In this study, we explored the potential mechanism of xylem differentiation by investigating the accumulation of miRNAs and mRNAs in different xylem tissues. Through the construction of a regulatory network between differentially accumulating miRNAs and their target genes, we obtained valuable miRNA-mRNA pairs involved in xylem differentiation and identified their interaction and potential role. These data provide a deeper understanding of the molecular mechanism of wood formation in *D. odorifera*.

### mRNA Sequencing Analysis

By analyzing the patterns of mRNA accumulations in two tissue types related to wood formation in *D. odorifera*, we identified 395 differentially expressed mRNAs between Dotz and Dosw (Figure 1A). Further, the enriched GO terms associated with the DEGs were largely expected. Anion transport and inorganic anion transport were highly enriched, indicating the function of these genes in wood formation. KEGG analysis showed that DEGs were commonly enriched in some pathways, including “carotenoid biosynthesis,” “diterpenoid biosynthesis,” and “alpha-linolenic acid metabolism”. This result is also consistent with our expectations as these three pathways might play an important role in the differentiation of xylem, especially in the biosynthesis of terpenoids and plant hormones (Biesgen and Weiler, 1999; Regnault et al., 2014).

We have identified genes that are possibly involved xylem differentiation process. Eleven genes that may encode transcription factors were identified, eight of which belong to the WRKY family. Our findings on differential expression of many genes encoding transcription factors are supported by previous investigations on other trees, such as rubber tree (Meng et al., 2021) and *Populus tomentosa* (Chen et al., 2015). In *P. tomentosa*, some genes of WRKY family were also differentially accumulated amoung the xylem tissues of tension wood, opposite wood, and normal wood (Chen et al., 2015). Further, earlier studies have shown that WRKY transcription factors were widely involved in plant responses to biotic, abiotic, and hormonal stresses, and regulate the biosynthesis of phenols, terpenes, and alkaloids (Schluttenhofer and Yuan, 2015). For example, *MdWRKY11* promotes the expression of *F3H*, *FLS*, *DFR*, *ANS,* and *10UFGT* in *Malus domestica* callus, and then increases the accumulation of flavonoids and anthocyanins (Wang N et al., 2018). Further, we also found some genes (such as *PER47*, *COMT*, *CCR2*, *UXS2*, *KAO2*, *GA20OX1*, *OPR2*, *CYP74A*, *PAL2*, and *CYP71A1*) related to lignin synthesis and cell wall composition, gibberellin synthesis, jasmonic acid synthesis, and flavonoids and terpenoids synthesis. Similarly, in the study of Sun et al. (2020), seven PAL family genes involved in the phenylpropane pathway were also significantly differentially expressed between the discolored wood by pruning and the normal wood in *D. odorifera*. In addition, *COMT* silenced transgenic poplar trees have significantly reduced 17% lignin levels (Jouanin et al., 2000). The trunk dry mass of a three-month-old *PdGA20ox1* overexpressing transgenic poplar was four times higher than that of untransformed control poplar; the contents of xylose and glucose were also significantly increased in these transgenic plants (Park et al., 2015).

### MiRNA Sequencing Analysis

MiRNAs are an important class of non-coding factors that regulate genes expression at the post-transcriptional level in nearly every aspect of plant development such as germination, growth, tissue differentiation, and flowering (Das et al., 2019; Glazińska et al., 2019; Qiu et al., 2019). The results of small RNA-seq demonstrate that the xylem of *D. odorifera* contains a large and diverse small RNA population, a finding similar to previous studies in rubber tree (Meng et al., 2022) and Chinese fir (Wan et al., 2012).

A total of 40 known miRNAs belonging to 22 miRNA families and 123 novel miRNAs were identified in these small RNA libraries, among which 14 miRNAs were differentially expressed (Figure 4). Similarly, the miR156, miR159, miR166, miR319, miR396, miR398, and miR408 families have also been identified in the xylem of rubber tree (Meng et al., 2022). The miR396a and miR156g are also differentially expressed in the primary stem, transition stem, and secondary stem of *Populus trichocarpa* (Wang R et al., 2021). Then, we found many miRNA families in *D. odorifera*, for example, miR156 (Wang et al., 2011), miR166 (Chen et al., 2018), and miR397 (Lu et al., 2013), are known to play a role in wood formation in other species. Subsequently, functional analysis of the target genes of the differentially expressed miRNAs revealed that these genes were commonly enriched in a few pathways including, “protein processing in endoplasmic reticulum,” “fatty acid elongation” and “biosynthesis of unsaturated fatty acids” These results further suggest that these differentially expressed miRNAs possibly regulate xylem differentiation of *D. odorifera*.

### Integration Analysis of Differentially Expressed miRNAs and Target Genes

We predicted 1,267 target-miRNA pairs between the 14 differentially expressed miRNAs and 1,056 candidate genes with the help of psRobot in Dotz and Dosw. Among these miRNA target related gene pairs, 22 showed a significantly negative correlation. Some studies have found that miR156 regulates plant growth and development, morphogenesis, anthocyanin accumulation, gibberellin synthesis, and stress response by inhibiting the expression of SPL transcription factors in *A. thaliana* at the post-transcriptional level (Schwarz et al., 2008; Jung et al., 2011). In our study, miR156g appears to negatively regulate seven target genes, including *SPL6* and *SPL12*. Novel miRNAs were equally differentially accumulated and significantly negatively correlated to their target genes, thus indicating an important regulatory role in xylem differentiation. In our study, the novel_130 is predicted to target the expression of up-regulated *GCS1* in Dotz. In *A. thaliana*, α-Glucosidase I (encoded by *GCS1*) is required for cellulose biosynthesis and morphogenesis (Gillmor et al., 2002). These results indicate that novel_130 may affect cellulose synthesis in the Dotz of *D. odorifera* by regulating *GCS1*.

## Conclusion

In summary, mRNA and small RNA profiles were first revealed in the process of xylem differentiation of *D. odorifera*. A total of 395 differentially expressed mRNAs were identified, many of which are involved in diterpenoid biosynthesis and alpha-linolenic acid metabolism, and controlled synthesis of terpenoids. Further, eight genes encoding the WRKY transcription factors, and some genes related to lignin synthesis, cell wall composition, gibberellin synthesis, jasmonic acid synthesis, flavonoids, and terpenoids synthesis (such as *PER47*, *COMT*, *CCR2*, *UXS2*, *KAO2*, *GA20OX1*, *OPR2*, *CYP74A*, *PAL2*, and *CYP71A1*), were also identified. Subsequently, 14 differentially expressed miRNAs between Dotz and Dosw were found, and nine of these were significantly negatively correlated to the expression of 21 target genes. This evidence provides valuable information for further functional characterization of the miRNAs and their targets in the xylem differentiation of *D. odorifera*.

## Data Availability

The small RNA and transcriptome data for *D. odorifera* reported in this paper has been deposited at the Genome Sequence Archive (Wang et al., 2017) in BIG Data Center (BIG Data Center Members, 2019), Beijing Institute of Genomics (BIG), Chinese Academy of Sciences, under accession numbers CRA006116 and CRA006117, and are publicly available at https://bigd.big.ac.cn/gsa.
