# Supplementary material for: Integrated mRNA and Small RNA Sequencing Reveals microRNAs Associated With Xylem Development in Dalbergia odorifera
Source: Front Genet. 2022 Apr 25;13:883422. doi: 10.3389/fgene.2022.883422 (PMC9081728; doi:10.3389/fgene.2022.883422)
Supplement: Supplementary file 1 [file DataSheet2.pdf]

Supplementary Table S1 Oligonucleotide primers used in real-time PCR assays in this study

Gene name	Forward primer	Reverse primer
Actin	CAATGAATTGCGTGTTGCT	ATACCAGTTGTGCGACCACTT
U6	CCTTCGGGGACATCCGATAAAA	GCAGGGGCCATGCTAATCTTCT
WRKY22	CAACCACCCTGCTCCCACT	CTCCACCTCATCATCATCCATC
AP2	CTCATCGCCGAGGATAATAACA	CGAGAATAACCGAACAACAACG
FPP7	CGAGAATAACCGAACAACAACG	CGAGAATAACCGAACAACAACG
PAL2	TCAGCTTTCTTCACTTTCGTTGC	GGTACTCGTCTACCATGCGTTTC
SPL12	ATCAGCACATAACGAATCAGCG	CAGACTAGCCAACAAAATAAGGGA
ARF8	GCACCTATACAGGTCAATGCCT	GGGTCAACTTGCCCTCCACT
FH20	GGGCTGTTGTTGTAAGGGTCT	ACTCACGGTTTGTAGCAGGTAATA
GCS1	GACATTCTGACGGAGGCGGC	GTCCATAATCTTCGGGGCGG
MMT1	AACGACGGATACCAAGGGAG	AGGCAACCACTTCTCAGCAA
miR156g	GTTGACAGAAGATAGAGAGCAC	
miR167e	TGAAGCTGCCAGCATGATCTG	
miR168a-5p	TCGCTTGGTGCAGGTCGGGAA	
novel_9	AAGCTGCCAGCATGATCTGAGA	
novel_15	TGCCAAAGGAGAGTTGCCCTG	
novel_52	CGTGGAAGTACTTTGAGGATCA	

Supplementary Table S3 Read count of known mature miRNAs

miRNA	Dotz1	Dotz2	Dotz3	Dosw1	Dosw2	Dosw3
miR156a	7	20	0	2	47	4
miR156g	3	4	1	5	13	1
miR159a	60698	92066	42200	1167	75778	13188
miR159c	0	0	0	0	2	0
miR159d	11	16	47	0	12	1
miR160a	1	0	0	0	0	1
miR162a	1289	5333	1074	61	3058	661
miR164a	153	377	194	11	384	157
miR166a	3	21	10	2	8	9
miR166n	8	24	5	4	10	4
miR167a	3	4	31	0	2	0
miR167e	7155	5948	20030	7	539	18
miR167f-5p	5	4	32	0	1	0
miR167h-5p	2	14	16	0	0	0
miR168a-3p	65	27	212	12	56	18
miR168a-5p	317	276	351	8	160	21
miR171a-3p	1	0	3	0	0	2
miR172a	0	3	4	2	3	0
miR172d	0	0	0	2	0	1
miR172g-3p	0	27	0	2	13	1
miR319a	1589	5191	2209	392	4690	2842
miR319e	18	51	38	28	43	25
miR319i	0	2	0	0	0	0
miR390a	1	5	24	13	173	680
miR393a-3p	2	0	1	0	4	0
miR393a-5p	0	1	0	0	0	0
miR394a-5p	361	1998	1104	20	920	227
miR395b	0	1	0	0	1	9
miR396a	10191	22199	4708	344	24556	7210
miR396c	4634	3950	3974	103	3534	1057
miR396e-3p	7	3	74	0	10	5
miR396f	4	4	1	1	11	0
miR396g-5p	0	10	2	0	13	4
miR397a	3	9	4	0	5	0
miR397b	1	9	4	0	4	0
miR398b	8	5	47	0	31	17
miR399h	1	0	0	0	0	0
miR403a	1	1	4	0	1	1
miR408-3p	2	2	1	0	2	1
miR6478	396	1239	965	438	838	477

Supplementary Table S4 Base type of each position of the known miRNAs

position	Dosw1				Dosw2				Dosw3				Dotz1				Dotz2				Dotz3			
	A	U	C	G	A	U	C	G	A	U	C	G	A	U	C	G	A	U	C	G	A	U	C	G
1	12	2026	479	49	185	111256	1109	370	683	24800	594	146	12	83206	554	287	18	134796	1439	329	35	75217	1266	284
2	15	1933	521	97	198	107326	4182	1214	631	24003	1210	379	17	74561	2102	7379	47	123008	6927	6600	42	53565	2660	20535
3	16	1175	377	998	639	70697	25739	15845	38	12424	7776	5985	7176	57830	11696	7357	6025	85563	23620	21374	20085	36540	8235	11942
4	578	19	432	1537	5208	57	26307	81348	1474	71	8472	16206	9172	11	12185	62691	13266	33	24142	99141	22563	32	8589	45618
5	785	101	530	1150	36136	3477	2031	71276	11534	1344	892	12453	16673	1648	910	64828	35797	5682	3492	91611	16307	1517	2492	56486
6	1269	129	1160	8	75038	6074	31704	104	13506	1170	11522	25	59629	3692	20711	27	93249	7490	35787	56	38868	6731	31167	36
7	468	2008	18	72	29299	82937	284	400	9155	16759	107	202	13069	70474	118	398	29297	106572	211	502	9314	66725	127	636
8	195	1591	5	775	3790	72155	60	36915	964	13014	19	12226	1764	58330	35	23930	5998	88506	48	42030	1631	38544	31	36596
9	919	26	461	1160	11537	323	30462	70598	4481	80	8744	12918	5578	365	20502	57614	13698	369	37156	85359	9523	375	30436	36468
10	1590	381	84	511	81345	26499	3720	1356	16893	7919	710	701	62710	11908	8565	876	97844	25489	11417	1832	44772	9066	21356	1608
11	1167	422	450	527	70895	28688	1204	12133	12307	8350	556	5010	64715	12878	835	5631	91190	28925	1653	14814	56478	9051	1398	9875
12	32	832	110	1592	233	27539	3491	81657	41	8433	825	16924	334	12490	1607	69628	319	26919	5670	103674	380	10290	1373	64759
13	66	110	831	1559	255	3533	27895	81237	169	861	8399	16794	68	1648	19515	62828	260	5722	32717	97883	244	1418	30255	44885
14	840	371	50	1305	11751	25758	1416	73995	4827	7760	432	13204	12411	11641	584	59423	19200	23635	2474	91273	29265	8043	1377	38117
15	1178	453	118	817	71674	26871	3221	11154	12730	8501	770	4222	58083	19349	1340	5287	87631	30142	5515	13294	37752	28647	1286	9117
16	76	495	433	1562	3380	1056	11673	96811	1327	597	4120	20179	1338	496	5686	76539	5407	1510	14687	114978	1121	1192	9950	64539
17	373	900	1204	89	26123	14149	71395	1253	7707	4865	12668	983	18709	6549	57907	894	29463	18626	87379	1114	28008	10298	37588	908
18	361	1173	511	521	25688	72155	13608	1469	7697	12675	5124	727	11660	65199	6132	1068	23622	93562	17493	1905	8026	57576	9395	1805
19	64	58	1944	477	666	5652	104754	1603	199	1018	23652	1299	516	3243	79269	872	525	6980	126856	1900	551	6042	68528	1498
20	170	1769	357	20	9086	96181	6192	159	1817	20153	3828	54	5074	75649	2142	107	12664	114732	7550	110	7731	64624	3344	122
21	1065	162	32	362	68405	2259	305	25923	12071	1622	709	7427	55722	2045	51	15594	82444	3017	123	31038	35635	4158	89	25284
22	13	93	2	6	573	300	117	20	25	499	15	5	6975	230	59	22	5850	488	105	20	19269	344	86	6

Supplementary Table S5 Read count of novel mature miRNAs

miRNA	Dosw1	Dosw2	Dosw3	Dotz1	Dotz2	Dotz3
novel_1	156	20658	6192	15251	35752	10980
novel_4	62	6139	4619	7242	8430	5345
novel_5	101	6046	1940	5148	9337	2404
novel_6	0	1	1	0	2	1
novel_8	57	2043	1232	1036	1840	355
novel_9	8	25	3	182	221	532
novel_10	8	1494	395	619	2108	896
novel_13	9	95	1184	3	4	5
novel_15	0	101	31	258	355	429
novel_18	3	52	39	48	146	107
novel_22	19	82	36	67	100	115
novel_23	0	38	9	46	119	18
novel_24	10	50	36	48	100	121
novel_26	1	27	107	10	26	26
novel_28	0	22	31	21	65	30
novel_30	11	50	27	30	60	55
novel_32	1	18	6	7	29	23
novel_34	0	50	9	1	43	10
novel_35	1	2	4	3	12	15
novel_36	0	2	1	6	13	4
novel_37	1	35	14	22	48	43
novel_38	0	15	12	16	41	14
novel_39	0	8	9	10	22	9
novel_40	1	4	9	7	13	13
novel_42	0	18	13	9	39	34
novel_44	0	15	16	3	22	9
novel_45	1	0	72	4	1	0
novel_46	8	22	21	22	16	6
novel_47	10	8	2	1	12	1
novel_49	0	2	20	1	2	5
novel_50	1	3	7	11	9	12
novel_51	0	20	6	5	22	28
novel_52	21	30	2	7	3	0
novel_54	1	11	2	9	10	11
novel_55	0	3	7	0	15	2
novel_56	0	6	3	11	6	1
novel_57	4	8	9	1	8	10
novel_58	1	10	14	4	19	19
novel_59	0	3	4	6	3	12
novel_60	1	9	3	7	17	27
novel_62	2	7	2	6	11	7
novel_64	0	13	0	10	22	15
novel_65	0	10	9	2	21	19
novel_67	0	13	0	6	14	1
novel_70	1	15	33	8	53	71

miRNA	Dosw1	Dosw2	Dosw3	Dotz1	Dotz2	Dotz3
novel_72	0	11	5	14	7	7
novel_74	2	28	21	39	45	68
novel_75	1	1	2	3	6	23
novel_76	4	39	11	10	36	31
novel_78	1	8	14	12	22	26
novel_79	2	12	16	2	8	17
novel_80	0	8	1	5	8	3
novel_81	0	5	1	3	18	9
novel_82	0	1	3	1	0	2
novel_83	0	8	8	5	7	23
novel_84	1	3	1	5	18	8
novel_85	1	11	16	0	29	2
novel_86	0	0	18	0	0	15
novel_87	0	38	23	10	27	37
novel_89	0	14	10	6	33	22
novel_90	2	10	23	26	42	46
novel_92	0	1	0	5	7	10
novel_93	0	10	5	8	26	8
novel_94	6	23	6	5	17	10
novel_95	0	5	0	1	2	1
novel_96	1	19	4	30	19	11
novel_97	2	6	18	5	8	3
novel_98	1	42	24	27	36	13
novel_99	9	50	53	36	74	29
novel_100	0	9	0	0	7	8
novel_101	0	11	6	5	8	1
novel_102	5	40	25	24	58	44
novel_103	0	6	0	7	20	5
novel_105	0	5	0	0	17	2
novel_107	0	18	4	8	39	10
novel_110	11	11	7	0	5	5
novel_111	0	16	3	0	21	5
novel_113	0	16	11	8	31	10
novel_114	0	2	0	1	19	3
novel_115	0	4	12	2	4	3
novel_116	0	13	3	12	5	12
novel_117	0	6	3	4	7	5
novel_118	0	32	9	5	83	25
novel_119	0	8	4	11	22	18
novel_120	3	6	3	8	17	9
novel_121	0	0	1	0	0	0
novel_122	0	12	9	4	18	17
novel_123	0	8	2	3	10	9
novel_125	1	2	15	56	8	50
novel_126	0	16	0	13	7	4
novel_128	0	5	1	22	18	2
novel_129	3	0	0	7	0	28

miRNA	Dosw1	Dosw2	Dosw3	Dotz1	Dotz2	Dotz3
novel_130	7	7	16	2	11	6
novel_131	0	14	2	0	11	5
novel_132	0	30	20	37	47	34
novel_133	7	111	69	60	275	108
novel_134	2	33	24	21	49	50
novel_136	0	5	0	5	20	17
novel_138	0	16	13	8	33	12
novel_139	6	45	36	21	56	32
novel_140	0	8	14	0	12	18
novel_144	0	22	2	31	28	11
novel_145	0	20	15	4	2	44
novel_146	3	10	11	4	26	25
novel_147	4	15	2	3	11	13
novel_148	0	17	3	20	34	17
novel_149	0	11	1	4	8	9
novel_150	3	0	28	38	0	77
novel_151	0	3	6	4	21	8
novel_152	24	27	0	16	20	0
novel_154	6	11	8	6	5	13
novel_155	0	12	15	23	17	78
novel_156	0	6	1	19	9	4
novel_157	0	25	0	12	61	5
novel_158	3	170	35	39	223	49
novel_159	6	9	9	69	15	11
novel_161	1	10	4	2	14	5
novel_162	0	3	3	5	11	3
novel_163	0	55	12	4	86	17
novel_164	1	15	3	16	9	6
novel_165	0	8	0	6	13	141
novel_166	1	11	4	0	15	4
novel_170	1	2	4	0	12	8

Supplementary Table S6 Base type of each position of the novel miRNAs

position	Dosw1				Dosw2				Dosw3				Dotz1				Dotz2				Dotz3			
	A	U	C	G	A	U	C	G	A	U	C	G	A	U	C	G	A	U	C	G	A	U	C	G
1	110	514	66	102	1319	37587	285	662	812	15126	1386	400	1079	30387	133	311	2239	59676	203	375	2056	21989	206	588
2	79	445	108	160	809	31280	6627	1137	471	10371	4997	1885	557	22587	7690	1076	1186	50623	9224	1460	845	15710	6121	2163
3	215	105	235	237	8890	1083	8610	21270	5852	702	4646	6524	8817	781	6725	15587	12190	1781	11998	36524	8171	1395	3633	11640
4	142	105	442	103	2400	1324	35286	843	1026	1903	14264	531	1343	685	29147	735	3113	1977	56175	1228	2214	1480	19924	1221
5	159	146	422	65	1205	2831	35108	709	1896	1193	14154	481	1065	1260	28808	777	1879	3580	55756	1278	1803	2073	19593	1370
6	275	113	71	333	8843	1158	2003	27849	3591	2003	647	11483	6898	883	1094	23035	12642	1483	2963	45405	3972	1796	1868	17203
7	404	186	54	148	30622	7428	617	1186	10137	5281	1561	745	22594	8168	487	661	49931	10614	768	1180	15509	7434	791	1105
8	85	468	54	185	1973	33932	510	3438	788	14524	478	1934	983	28406	420	2101	2867	54943	749	3934	1414	20097	797	2531
9	156	369	148	119	6861	27488	2916	2588	6229	8728	1825	942	7673	20978	1648	1611	9226	46372	3069	3826	6158	14494	1678	2509
10	135	118	428	111	6893	1021	29550	2389	4962	1819	9907	1036	7880	750	22101	1179	9603	1293	48305	3292	6573	1302	15120	1844
11	54	224	393	121	599	7257	29238	2759	392	5244	10999	1089	613	7908	21760	1629	1194	10140	47589	3570	1357	6609	14298	2575
12	309	141	68	274	29046	1103	783	8921	11675	723	1665	3661	23769	540	599	7002	47646	1292	1041	12514	18243	1013	1024	4559
13	102	163	429	98	2235	1418	35327	873	859	2079	14301	485	1202	813	29149	746	3142	1633	56299	1419	2089	1218	20286	1246
14	157	175	379	81	6882	2987	29392	592	5015	1426	9747	1536	7802	1692	22030	386	9735	4028	47970	760	6757	2762	14556	764
15	122	115	383	172	1061	1064	30630	7098	1763	749	10024	5188	627	1261	22342	7680	1427	1913	49608	9545	975	2219	15439	6206
16	472	130	34	156	36836	1017	602	1398	14663	695	388	1978	29595	721	420	1174	58308	1592	798	1795	20815	1214	660	2150
17	148	389	39	216	981	29734	1905	7233	631	11163	708	5222	923	22133	1091	7763	1714	48348	2844	9587	1965	14915	1664	6295
18	184	499	56	53	2609	35908	650	686	1084	15753	464	423	1300	29377	720	513	3349	56860	1148	1136	1789	20762	1262	1026
19	145	152	434	55	1186	1284	35395	1932	661	1980	14383	687	861	805	29337	858	1765	1346	56642	2658	1536	1175	20589	1515
20	91	148	453	79	965	2790	35072	800	703	1075	15360	527	605	1825	28790	557	1657	4176	55513	869	1259	3035	19565	802
21	134	388	66	138	1263	34268	792	2697	1837	13491	364	1563	801	28325	492	1747	1496	55575	1165	3325	1171	19972	898	2216
22	339	119	69	93	28418	6876	486	2094	9463	4972	434	767	21322	7563	461	1077	46650	9597	867	2973	14493	5965	696	1671

Supplementary Table S7 The differentially expressed miRNAs

miRNA	Dotz_readcount	Dosw_readcount	log_2FoldChange	pval
novel 110	1.71	24.70	-3.65	0.00
novel_130	3.41	19.90	-2.37	0.03
novel_15	217.75	32.77	2.65	0.01
novel 165	31.57	1.71	3.82	0.01
novel_45	1.41	27.85	-4.42	0.02
novel 47	2.25	20.45	-2.98	0.03
novel 52	2.65	44.96	-4.10	0.01
novel 9	194.54	20.84	3.37	0.00
miR156g	1.73	12.15	-2.66	0.04
miR167e	7076.17	134.15	5.71	0.00
miR167f-5p	8.47	0.21	4.03	0.02
miR167h-5p	5.82	0.00	4.36	0.02
miR168a-5p	209.62	56.16	1.88	0.02
miR390a	5.77	306.40	-5.72	0.00

Supplementary Table S8 Differentially expressed miRNAs and their target genes with significant negative correlations in expression

miRNA	target_mRNA_ID	target_gene_ID	gene_name	Target_start	Target_end	strand	score	Target_aligned_fragment	match	miRNA_aligned_fragment	Pearson correlation factor
miR168a-5p	evm.model.scaffold_5.541	evm.TU.scaffold_5.541	*MMT1*	253	272	+	6	UUUCUGACCUUCAC-AAGCGU	::.:.::::: ::: :::::	AAGGGCUGGACGUGGUUCGCU	-0.84
miR167h-5p	evm.model.scaffold_263.207	evm.TU.scaffold_263.207	*pyrH*	1794	1813	+	5.5	GAGAUCAAGUUGGCA-CUUUG	:::::: ::::::: :::..	GUCUAGUACAACCGUCGAAGU	-0.93
miR167f-5p	evm.model.scaffold_263.207	evm.TU.scaffold_263.207	*pyrH*	1794	1813	+	6	GAGAUCAAGUUGGCA-CUUUG	.:::::: :.::::: :::..	UUCUAGUACGACCGUCGAAGU	-0.95
miR156g	evm.model.scaffold_5.791	evm.TU.scaffold_5.791	*SPL12*	1847	1867	+	2	GUGCUCUCUCUCUUCUGUCAA	::::::::: :::::::::::	CACGAGAGAUAGAAGACAGUU	-0.82
miR156g	evm.model.scaffold_461.254	evm.TU.scaffold_461.254	*SPL6*	1211	1231	+	3	GUGCUCUCUCUCUUCUGUCAU	::::::::: ::::::::::	CACGAGAGAUAGAAGACAGUU	-0.93
miR156g	evm.model.scaffold_222.90	evm.TU.scaffold_222.90	*TSS*	3119	3138	+	4.5	GUGCUUUAUAUCUUCUG-CAU	:::::.: ::::::::: ::	CACGAGAGAUAGAAGACAGUU	-0.95
miR156g	evm.model.scaffold_384.206	evm.TU.scaffold_384.206	*KCR1*	1917	1937	+	5.5	ACUCUUUCUAUCUUCUGUUAC	::.::::::::::::.:	CACGAGAGAUAGAAGACAGUU	-0.98
miR156g	evm.model.scaffold_36.542	evm.TU.scaffold_36.542	*DCL3A*	3145	3164	+	5.5	UUGCGUUCUG-CUUCUGUCAA	::: .:::. ::::::::::	CACGAGAGAUAGAAGACAGUU	-0.92
miR156g	evm.model.scaffold_18.317	evm.TU.scaffold_18.317	*RIN2*	1001	1020	+	6	GUCCUCUCUG-CUUCUGUUAU	:: ::::::. ::::::::.:	CACGAGAGAUAGAAGACAGUU	-0.81
miR156g	evm.model.scaffold_394.168	evm.TU.scaffold_394.168	*T16L1_250*	49	68	+	6	CUGUUCUUCAUCUUCU-UCAA	::.:::. ::::::: ::::	CACGAGAGAUAGAAGACAGUU	-0.89
novel_9	evm.model.scaffold_35.394	evm.TU.scaffold_35.394	*ARF8*	1519	1541	+	4.5	CUUAGAUCAGGCUGGCAGCUUGU	::.:::::: :::::::::::	GAGUCUAGUACGACCGUCGAAGU	-0.85
novel_9	evm.model.scaffold_375.60	evm.TU.scaffold_375.60	*CPP1*	991	1012	+	5	CUCAUCUUA-GCUGGCAGCUUUG	:::: :.: ::::::::::::..	GAGUCUAGUACGACCGUCGAAGU	-0.84
novel_52	evm.model.scaffold_461.310	evm.TU.scaffold_461.310	*FH20*	623	643	+	4.5	UGAUUCUUAGAGUCCUUCCAU	::::.::.:.::: ::::::.	ACUAGGAGUUUCAUGAAGGUG	-0.94
novel_52	evm.model.scaffold_37.48	evm.TU.scaffold_37.48	*PMIR2*	3191	3210	+	5.5	GGAUCCUUAAAGUA-UACCAC	::::::.:::::: : ::::	ACUAGGAGUUUCAUGAAGGUG	-0.83
novel_52	evm.model.scaffold_205.104	evm.TU.scaffold_205.104	*ETL1*	1750	1770	+	6	CUGUCCUUGAAGUUCUUCCAC	.::::..:::: :::::::	ACUAGGAGUUUCAUGAAGGUG	-0.88
novel_52	evm.model.scaffold_419.150	evm.TU.scaffold_419.150	*PAP29*	1012	1031	+	6	UGGUUC-CAAAGAACUUCUGC	::.:.: ::::: :::::..:	ACUAGGAGUUUCAUGAAGGUG	-0.85
novel_52	evm.model.scaffold_39.645	evm.TU.scaffold_39.645	*VIP6*	2595	2614	+	6	AGACUCUCAAA-UACUUCUAU	:: .:::::: ::::::.:.	ACUAGGAGUUUCAUGAAGGUG	-0.82
novel_52	evm.model.scaffold_28.16	evm.TU.scaffold_28.16	*ANTR6*	1813	1833	+	6	UAAGCCUCAAAGUUUUUCCAA	: : ::::::::: .:::::	ACUAGGAGUUUCAUGAAGGUG	-0.85
novel_165	evm.model.scaffold_222.624.5	evm.TU.scaffold_222.624	*EZA1*	111	132	+	6	AACCUUGGUGAUGAAGAUCAGU	:: ::: :::::: .::::::	GUGCAAC-ACUACUCUUAGUCA	-0.93
novel_15	evm.model.scaffold_40.1112	evm.TU.scaffold_40.1112	*ACR9*	976	996	+	6	CAGGGCAUCUCUUCUCUGUCA	::::::: ::::.:: :: ::	GUCCCGUUGAGAGGAAACCGU	-0.84
novel_130	evm.model.scaffold_5.813	evm.TU.scaffold_5.813	*GCS1*	2105	2128	+	6	AUGGUGCUUACUUGGAUUUUGGAA	: : :. :.::::.::::::::.::	UUCAAUCAGUGAAUCUAAAACUUU	-0.85
novel_130	evm.model.scaffold_39.729	evm.TU.scaffold_39.729	*ERDJ2A*	2402	2426	+	6	AUGUUUGUGAGUUUGGAUUUUGAAG	: ::: :: : .::.:::::::::.	UUCAAUCAGU-GAAUCUAAAACUUU	-0.91
