# Supplementary material for: Integrated mRNA and Small RNA Sequencing Reveals microRNAs Associated With Xylem Development in Dalbergia odorifera
Source: Front Genet. 2022 Apr 25;13:883422. doi: 10.3389/fgene.2022.883422 (PMC9081728; doi:10.3389/fgene.2022.883422)
Supplement: Supplementary file 3 [file DataSheet1.pdf]

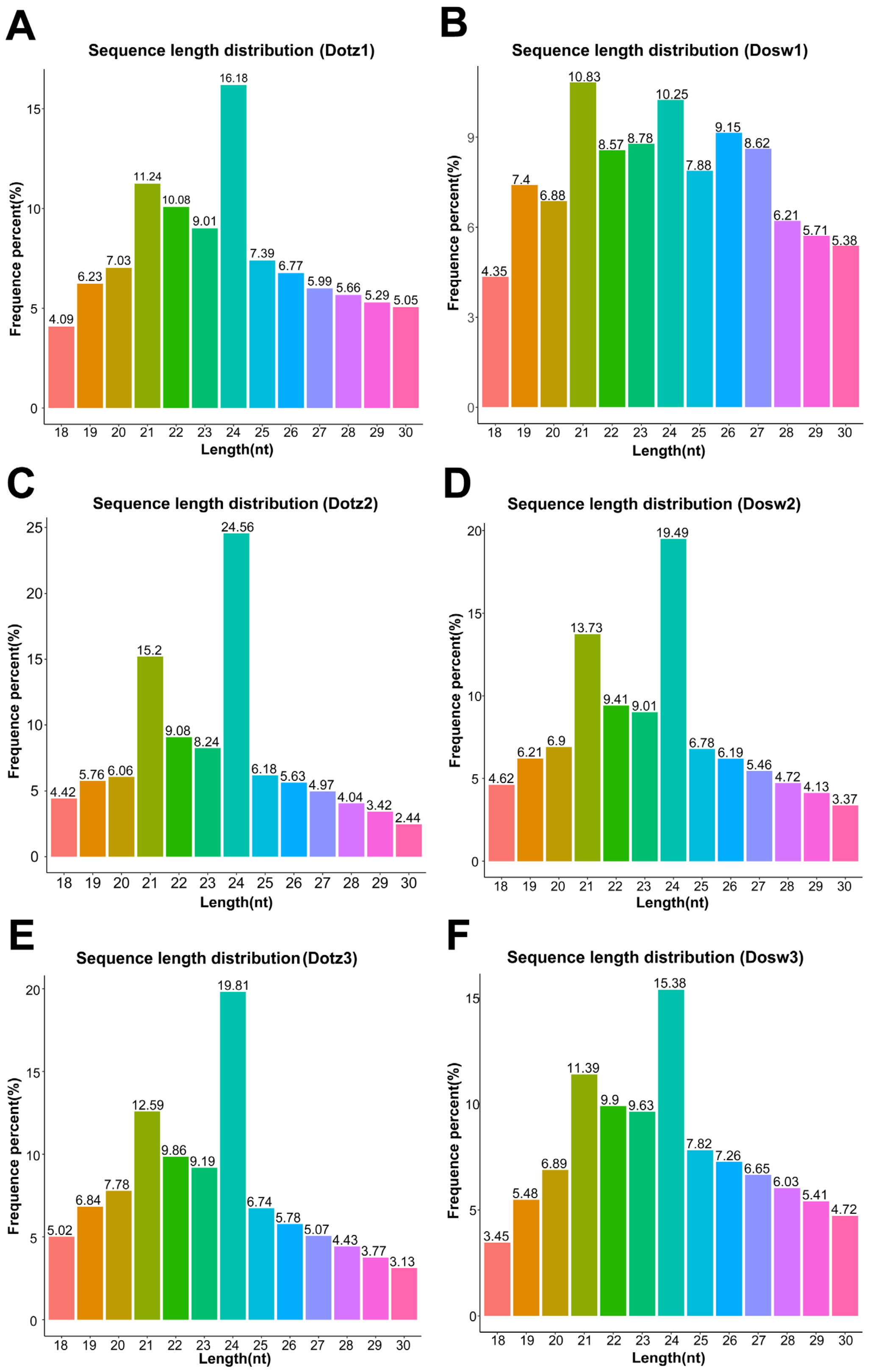

Supplementary Figure S1: Length distribution of all small RNAs sequences in the six samples. Dotz1 (A), Dosw1 (B), Dotz2 (C), Dosw2 (D), Dotz3 (E) and Dosw3 (F). The x-axis represents the length of small RNAs, and the y-axis shows the proportion of small RNA at any given length.

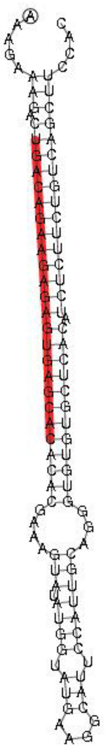

MIR156a

miR156a

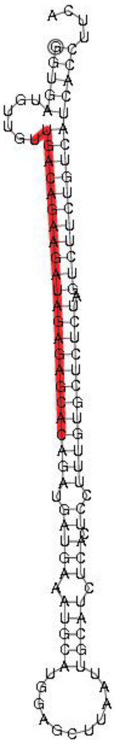

MIR156g

miR156g

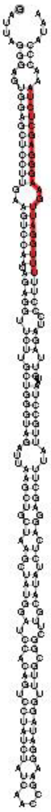

MIR159a

miR159a

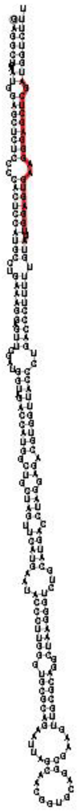

MIR159c

miR159c

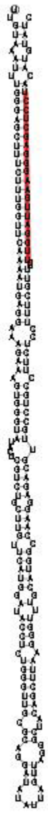

MIR159d

miR159d

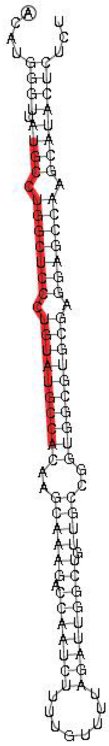

MIR160a

miR160a

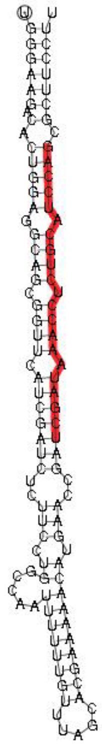

MIR162a

miR162a

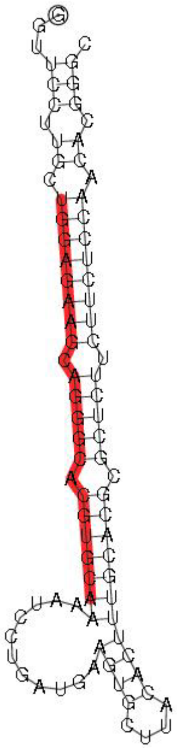

MIR164a

miR164a

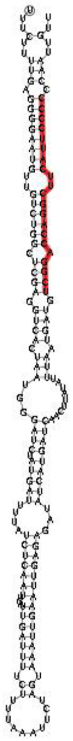

MIR166a

miR166a

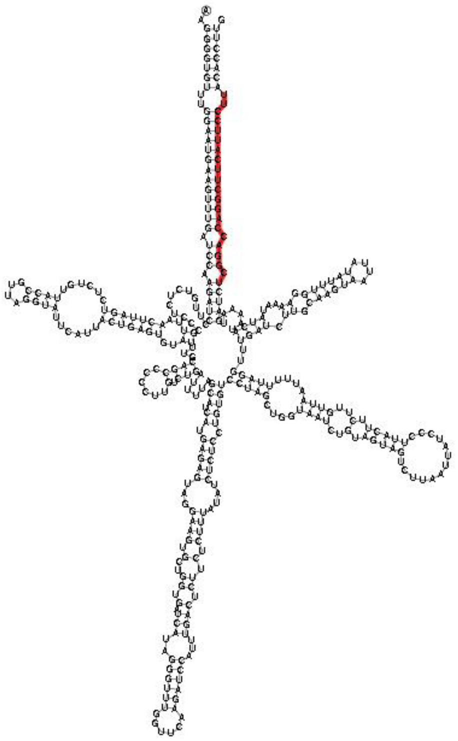

MIR166n

miR166n

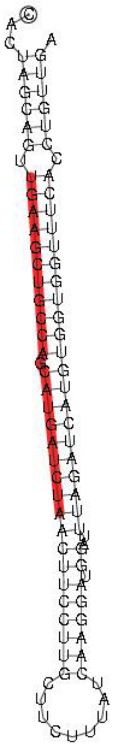

MIR167a

miR167a

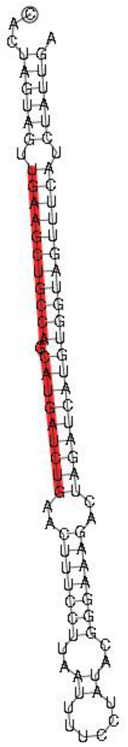

MIR167e

miR167e

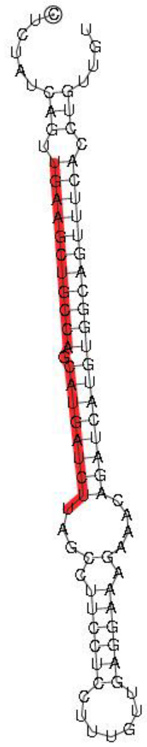

MIR167f

miR167f-5p

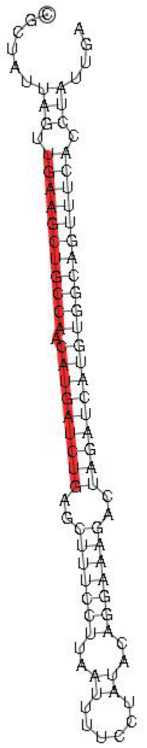

MIR167h

miR167h-5p

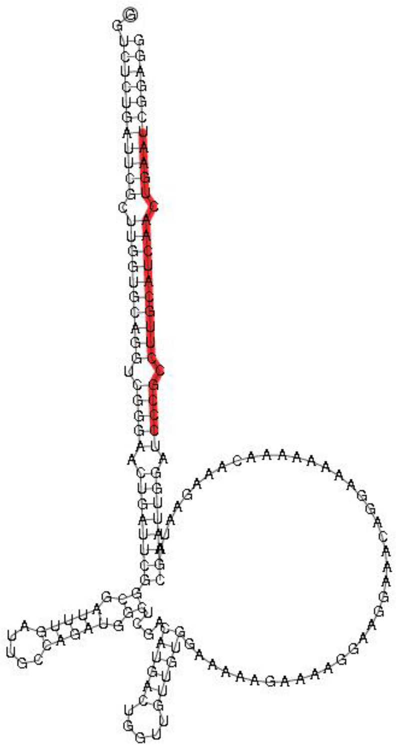

MIR168a

miR168a-3p

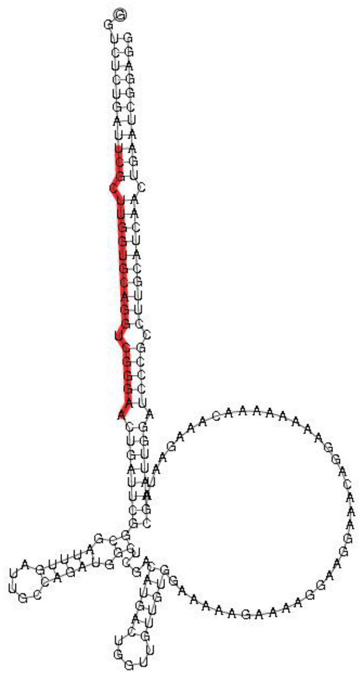

MIR168a

miR168a-3p

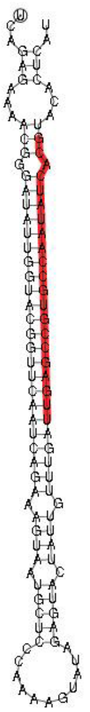

MIR171a

miR171a-3p

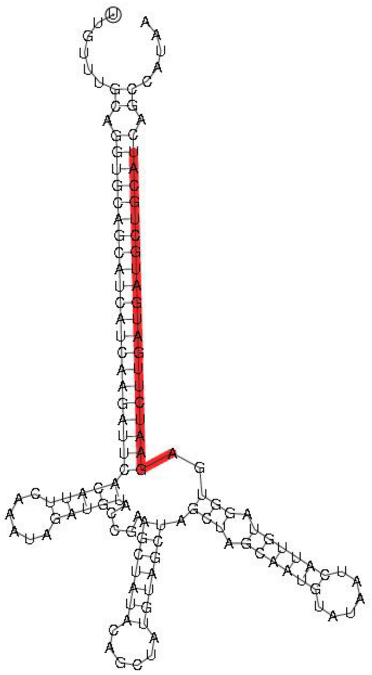

MIR172a

miR172a

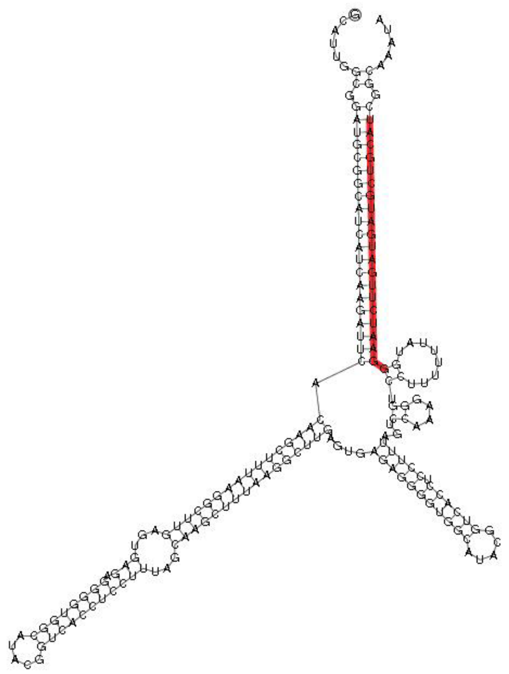

MIR172d

miR172d

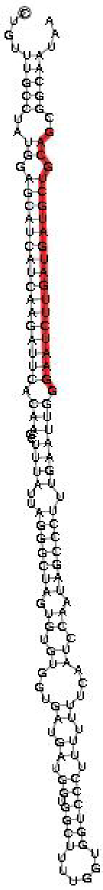

MIR172g
miR172g-3p

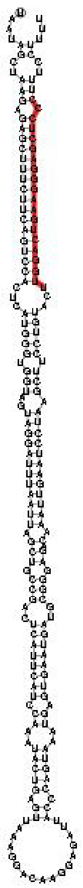

MIR319a
miR319a

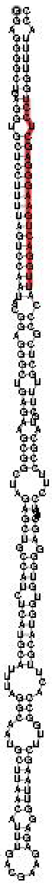

MIR319e
miR319e

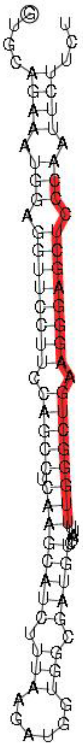

MIR319i
miR319i

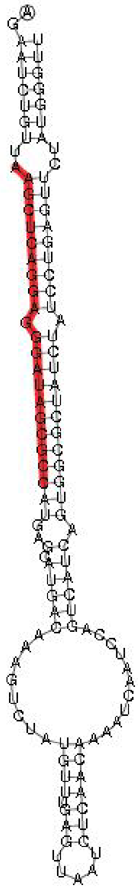

MIR390a
miR390a

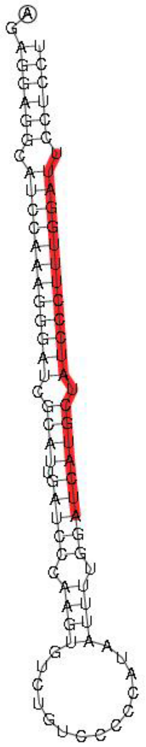

MIR393a
miR393a-3p

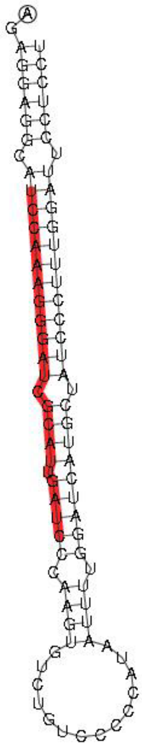

MIR393a
miR393a-5p

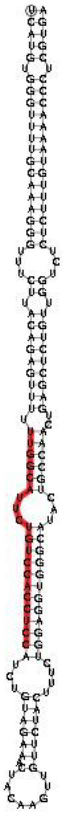

MIR394a
miR394a-5p

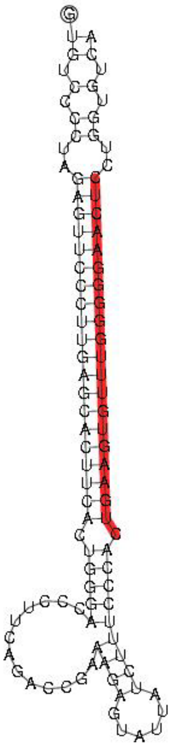

MIR395b
miR395b

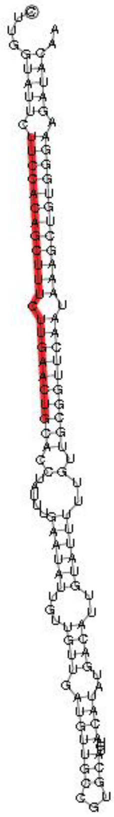

MIR396a
miR396a

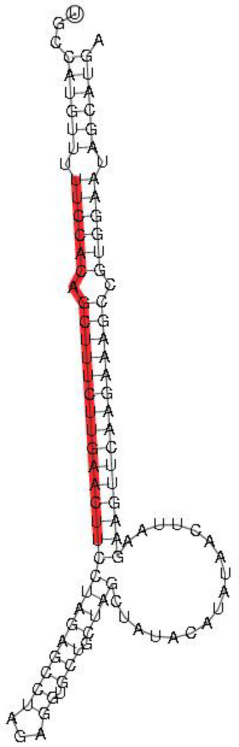

MIR396c
miR396c

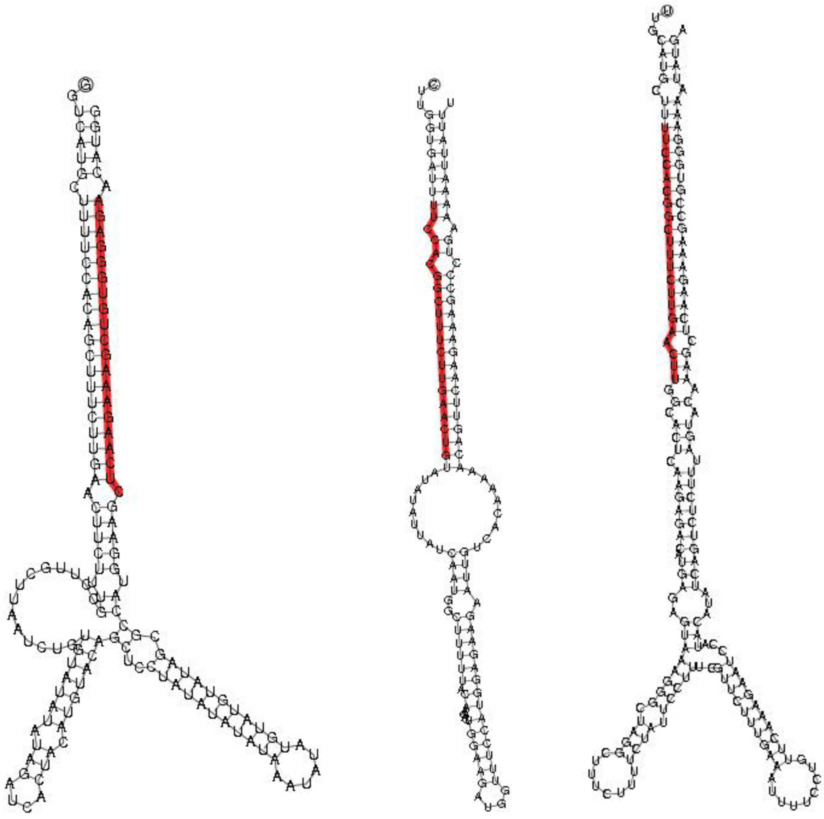

MIR396e miR396e-3p | MIR396f miR396f | MIR396g miR396g-5p | MIR397a miR397a | MIR397b miR397b

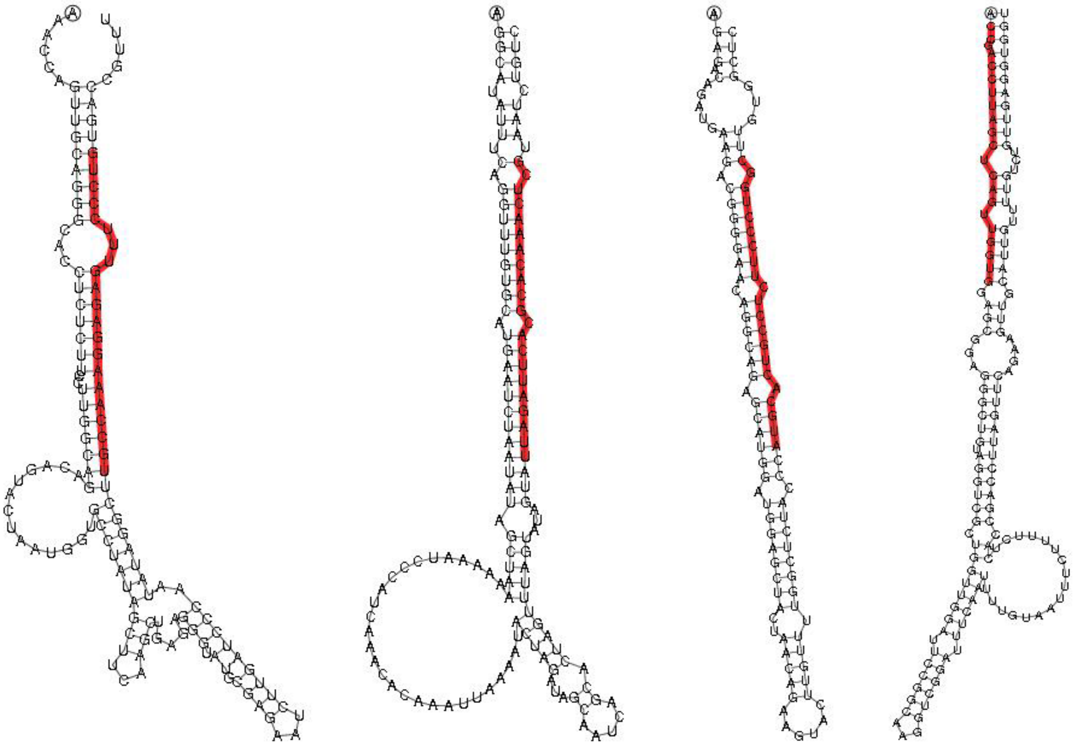

MIR398b miR398b | MIR399h miR399h | MIR403a miR403a | MIR408 miR408-3p | MIR6478 miR6478

Supplementary Figure S2: The predicted secondary structures of known miRNAs. The entire sequence is of the miRNA precursor, and the red highlight is the position of the mature miRNA.

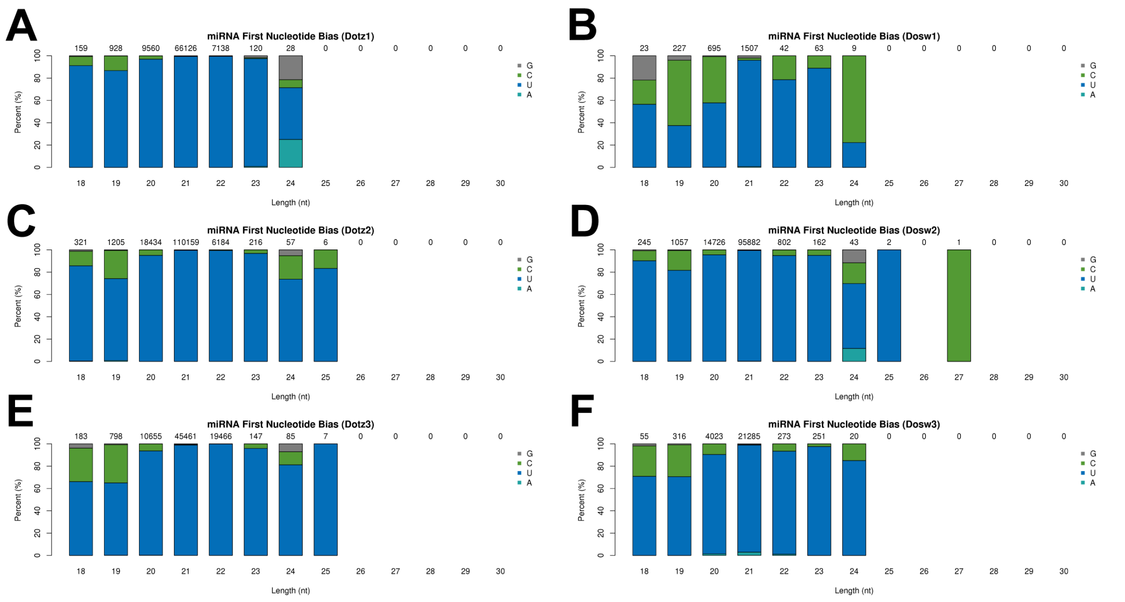

Supplementary Figure S3: First base composition of the known miRNA (18-30 nt) in six samples. Dotz1 (A), Dosw1 (B), Dotz2 (C), Dosw2 (D), Dotz3 (E) and Dosw3 (F). The x-axis represents the length of miRNAs, and the y-axis shows the percentage of A/U/C/G in the first base of miRNA at that length.

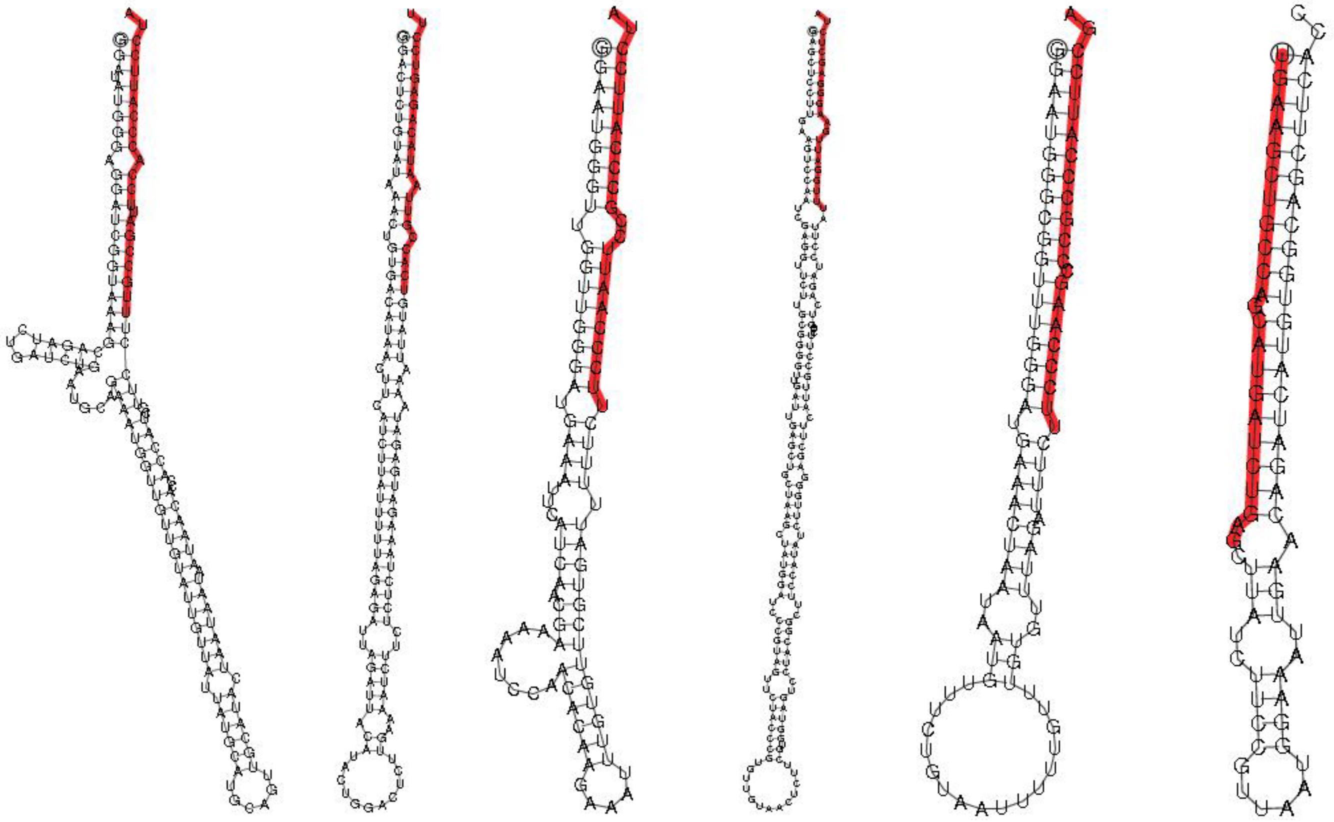

novel_1 novel_4 novel_5 novel_6 novel_8 novel_9

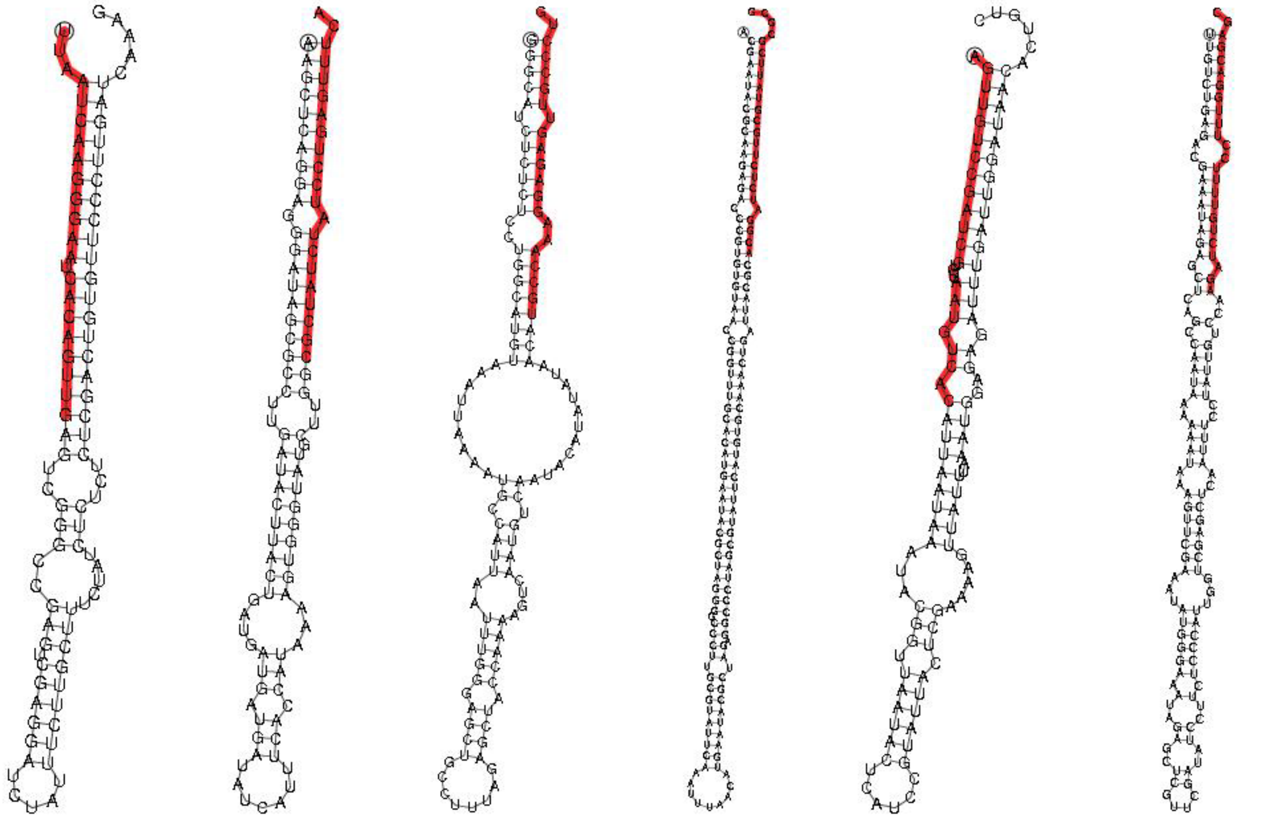

novel_10 novel_13 novel_15 novel_18 novel_22 novel_24

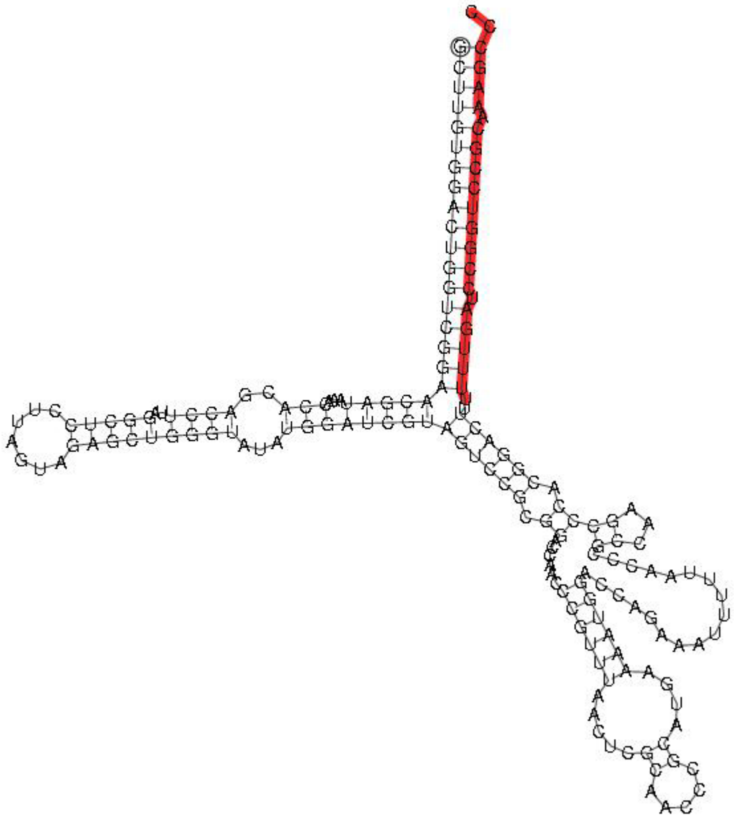

novel_23

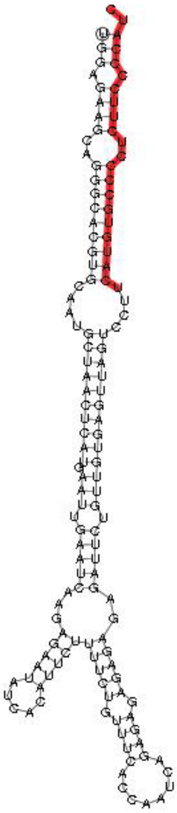

novel_26

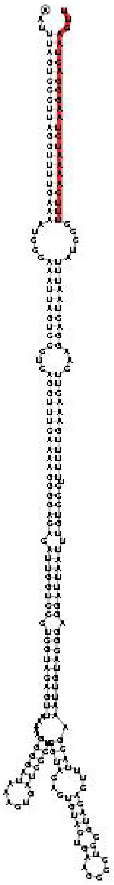

novel_28

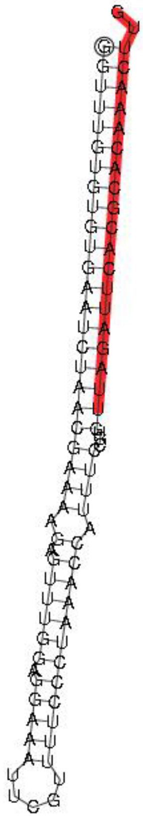

novel_30

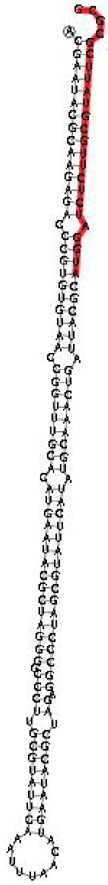

novel_32

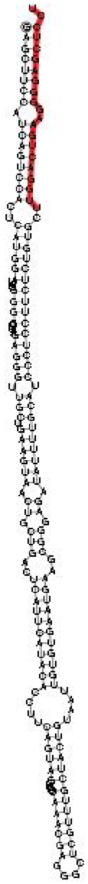

novel_34

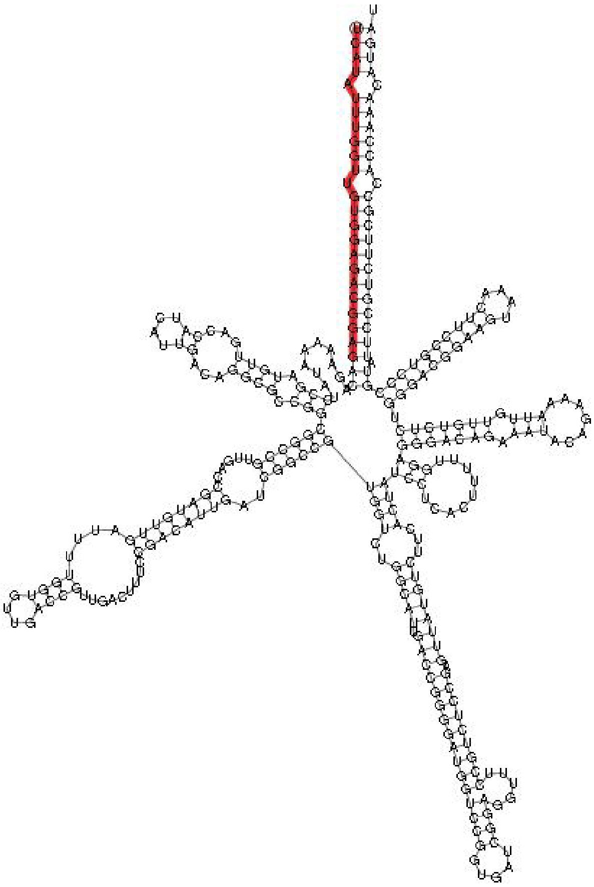

novel_35

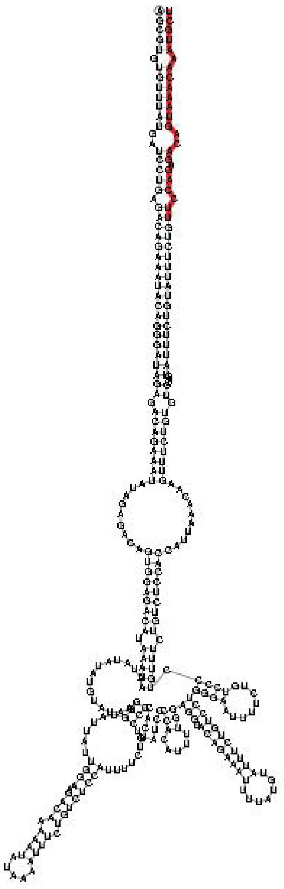

novel_36

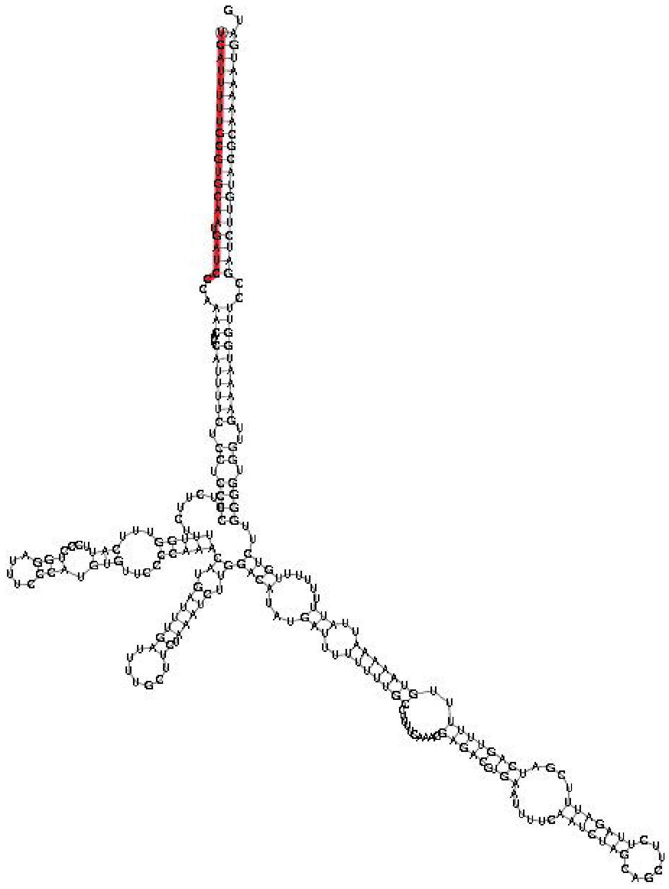

novel_37

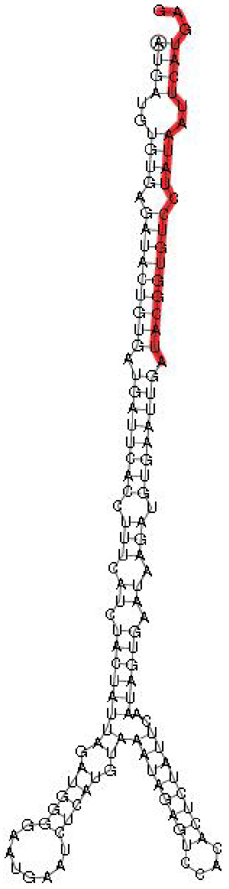

novel_38

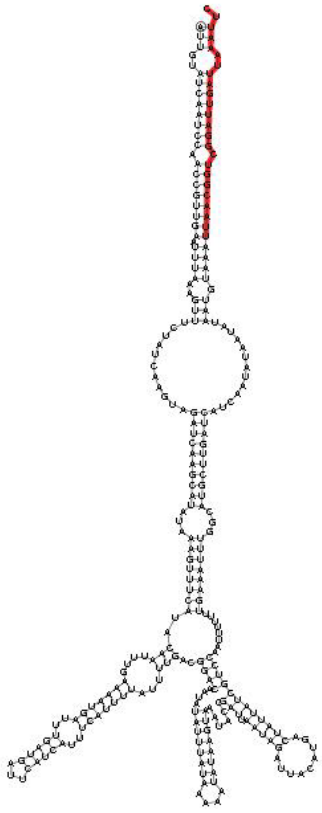

novel_39

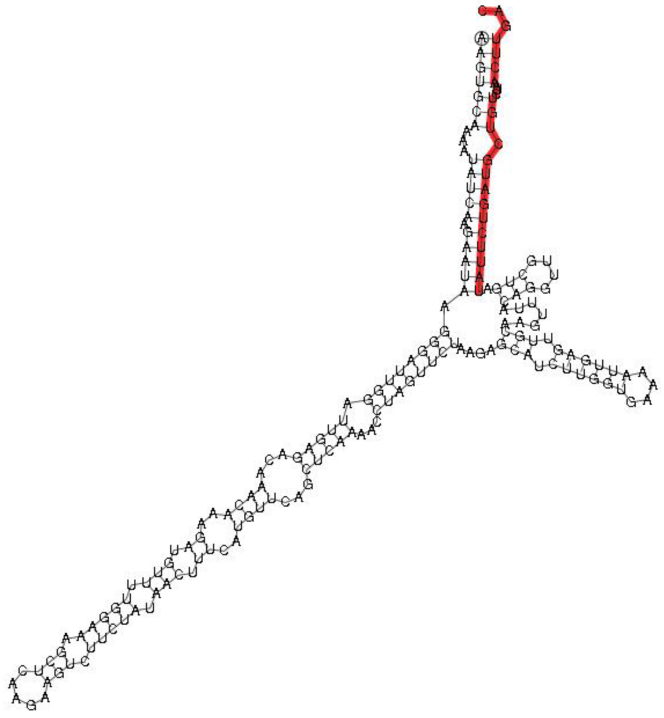

novel_40

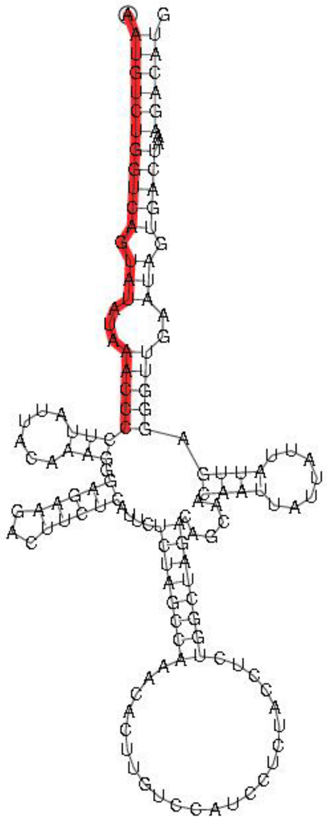

novel_42

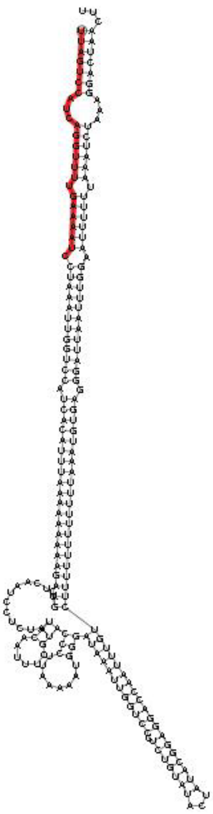

novel_44

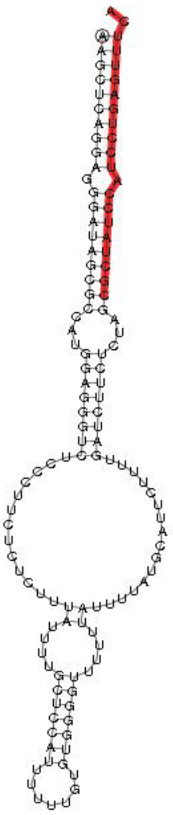

novel_45

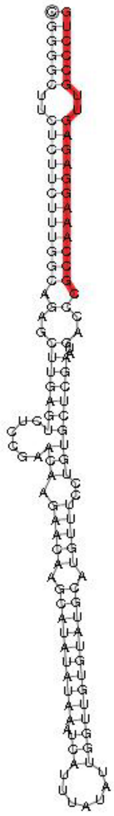

novel_46

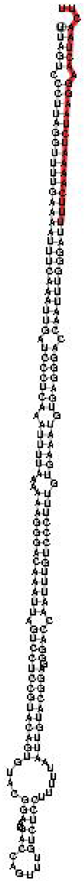

novel_47

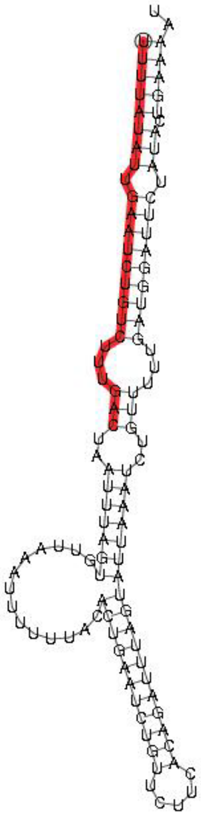

novel_49

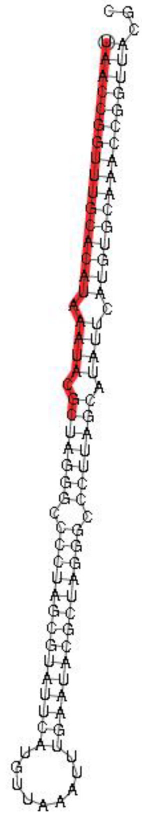

novel_50

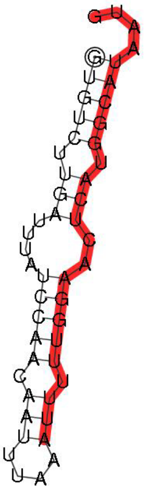

novel_51

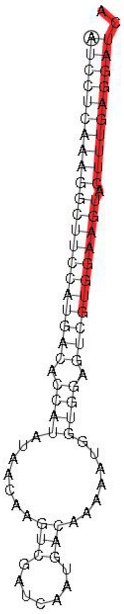

novel_52

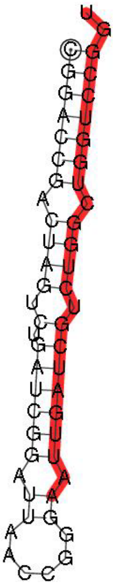

novel_54

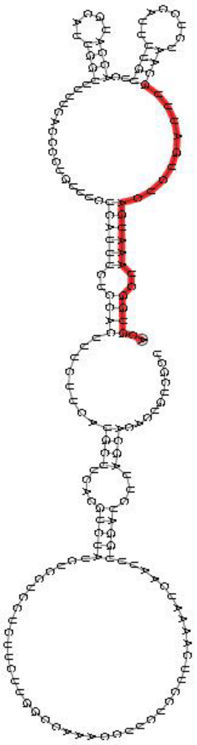

novel_55

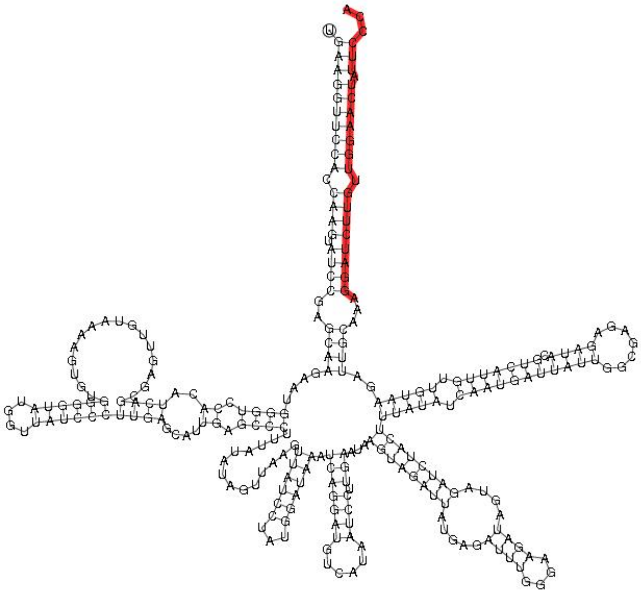

novel_56

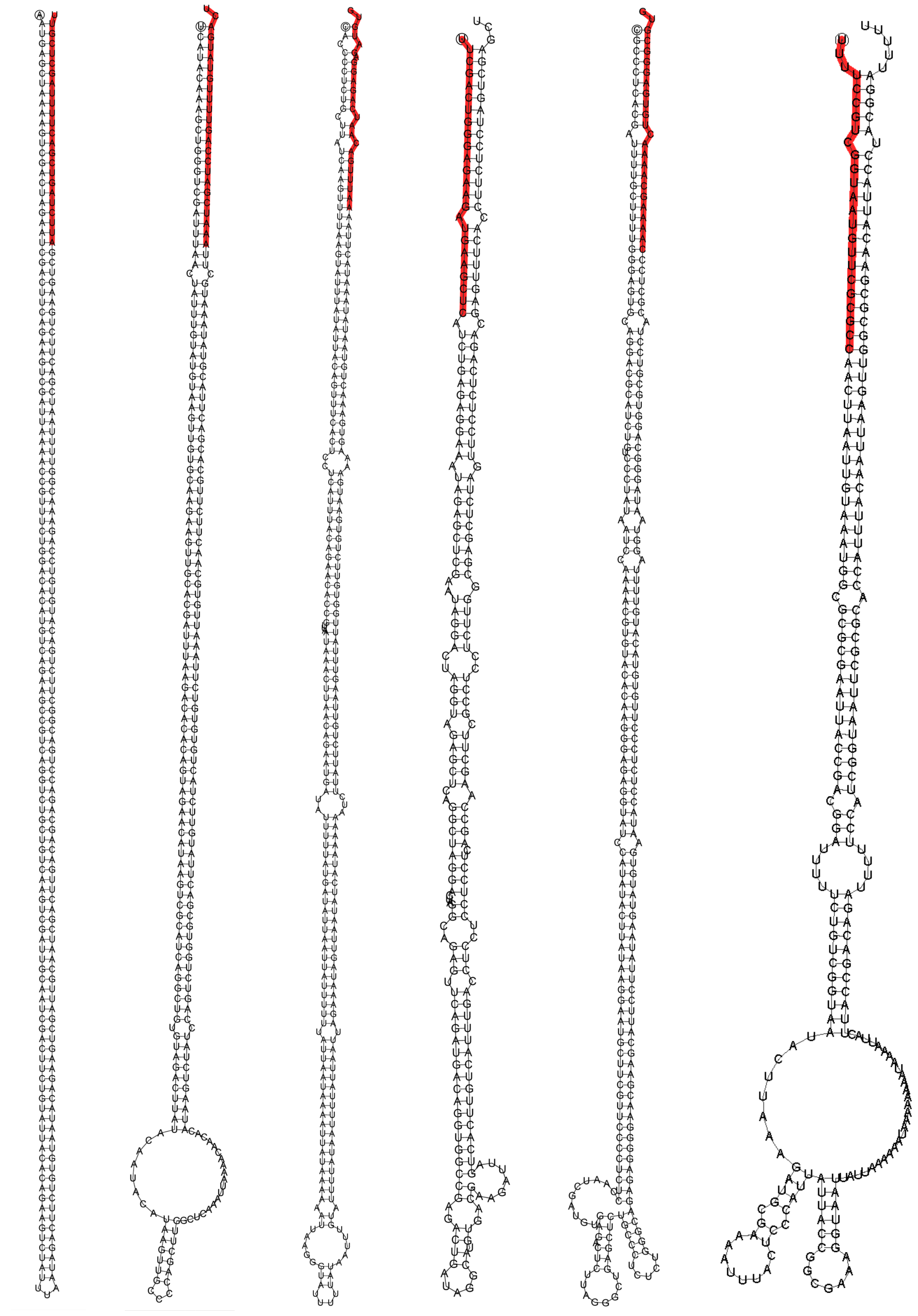

novel_57 novel_58 novel_59 novel_60 novel_62 novel_64

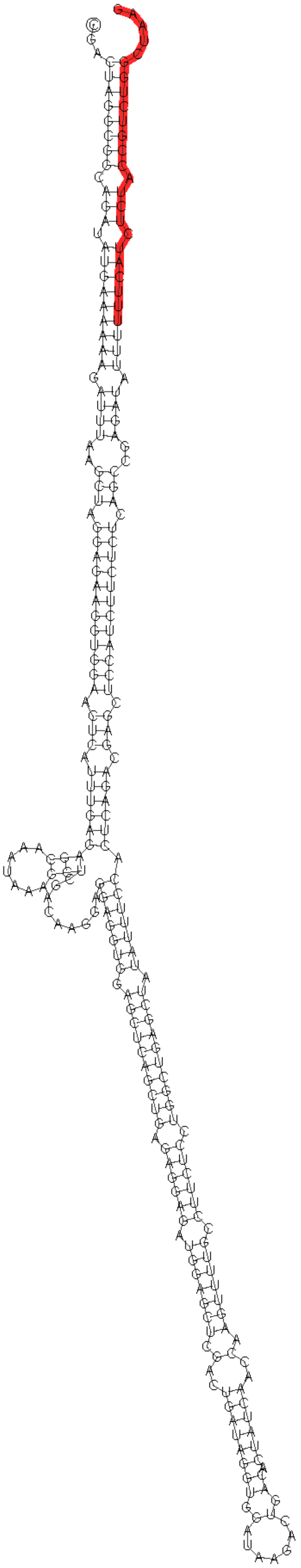

novel_65

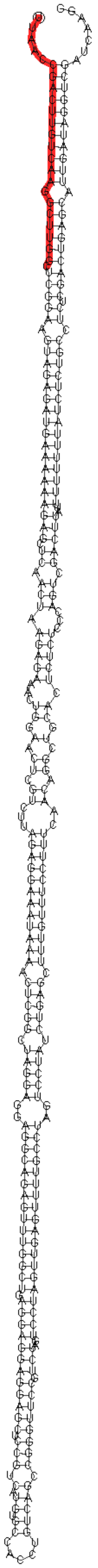

novel_67

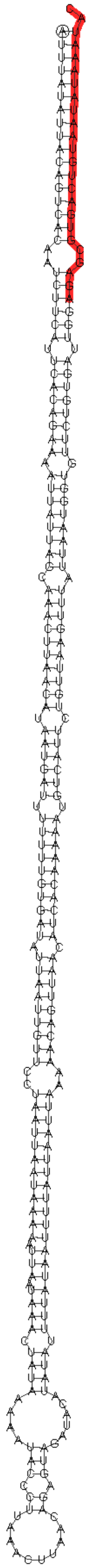

novel_70

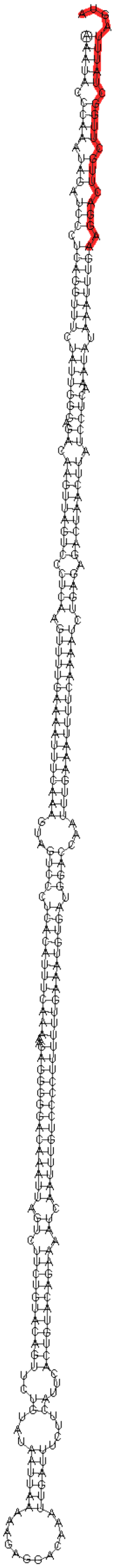

novel_72

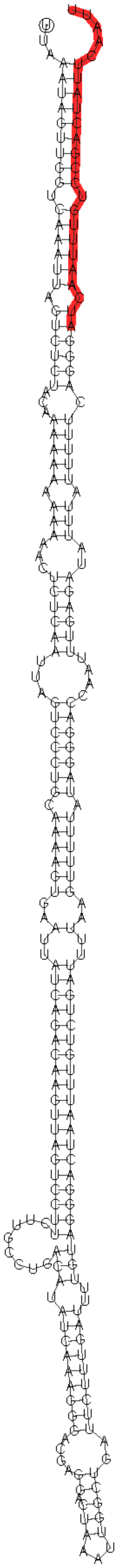

novel_74

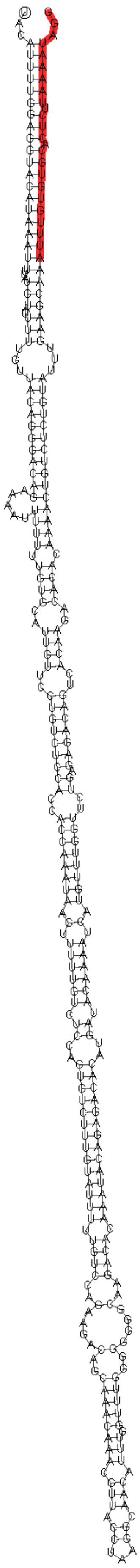

novel_81

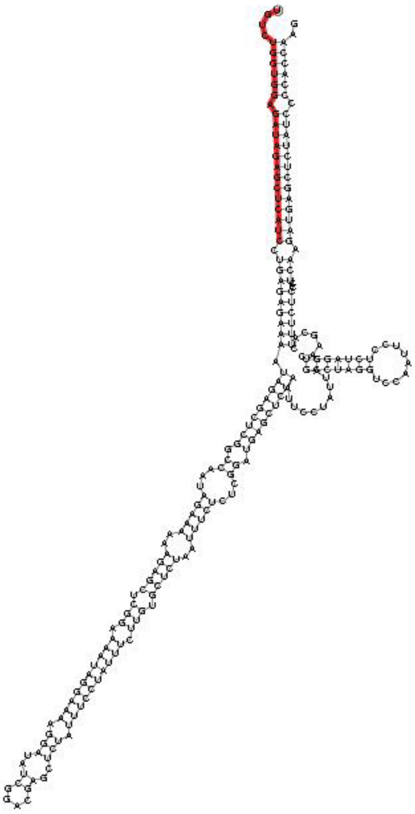

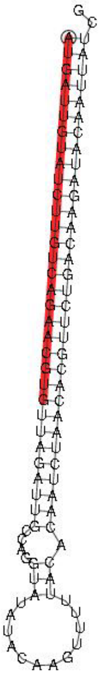

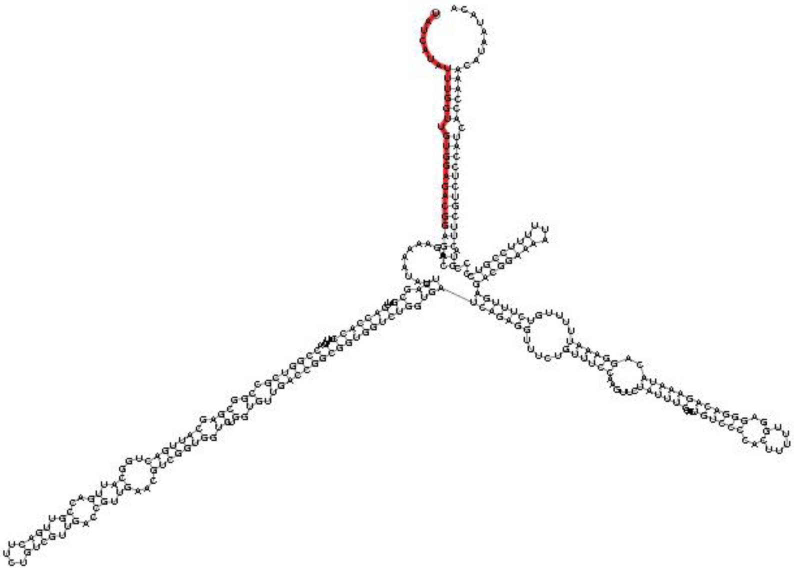

novel_75 **novel_76** **novel_78**

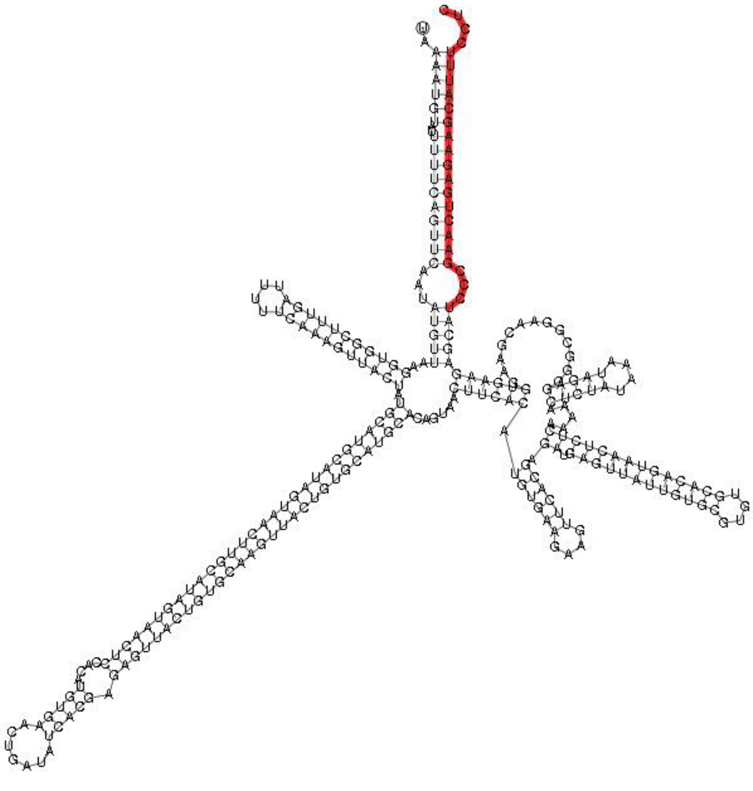

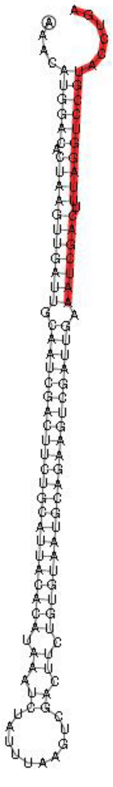

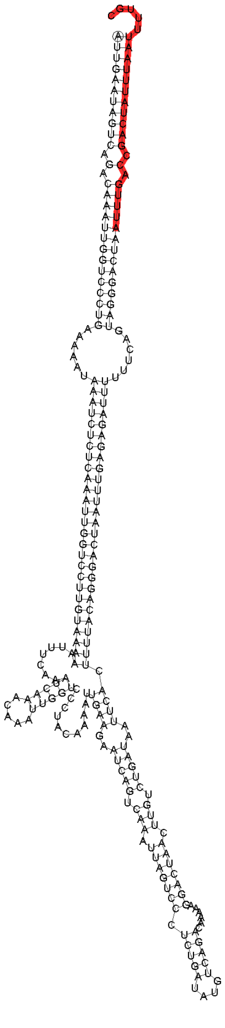

novel_79 **novel_80** **novel_82**

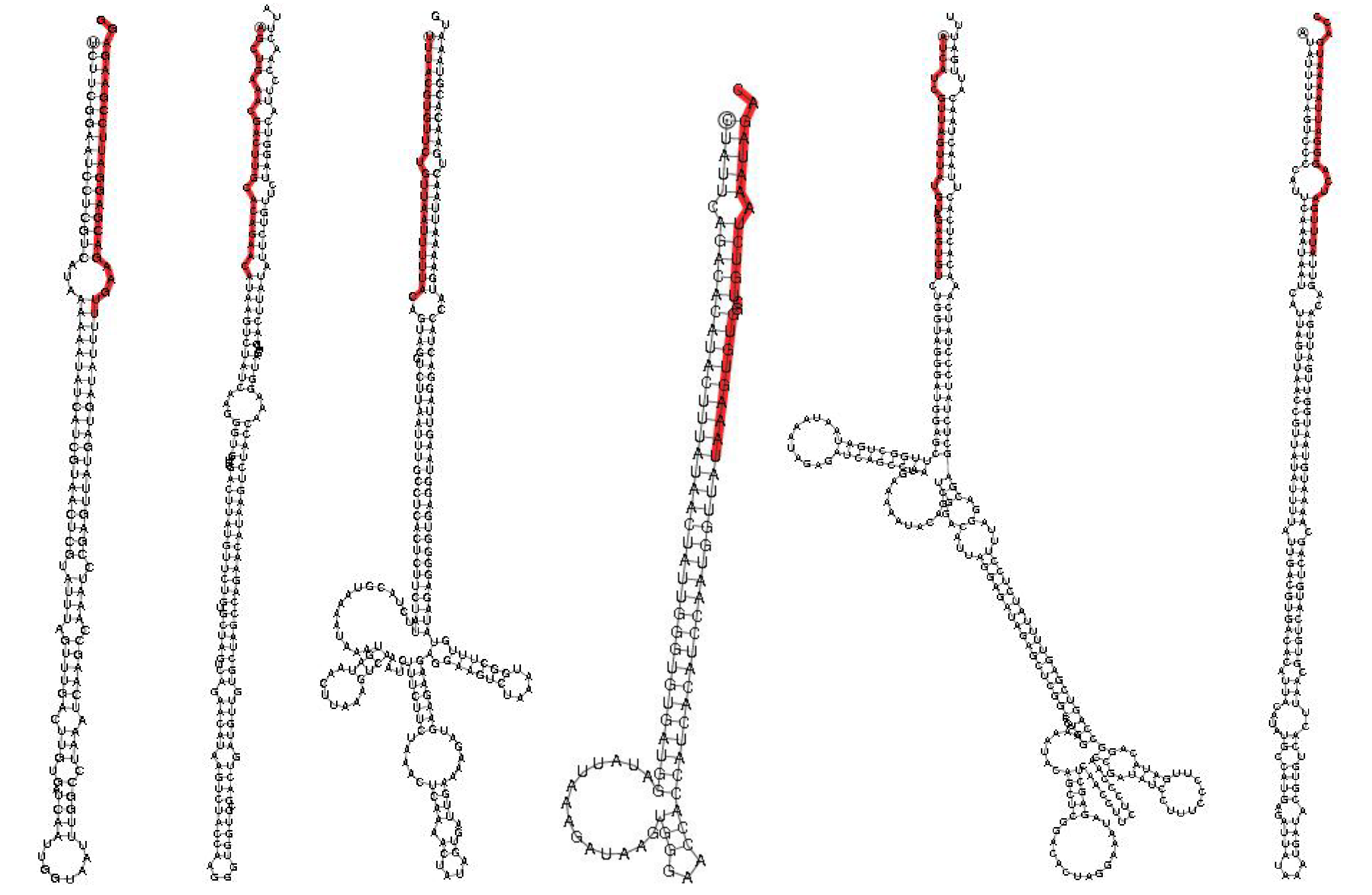

novel_83 novel_84 novel_85 novel_86 novel_87 novel_89

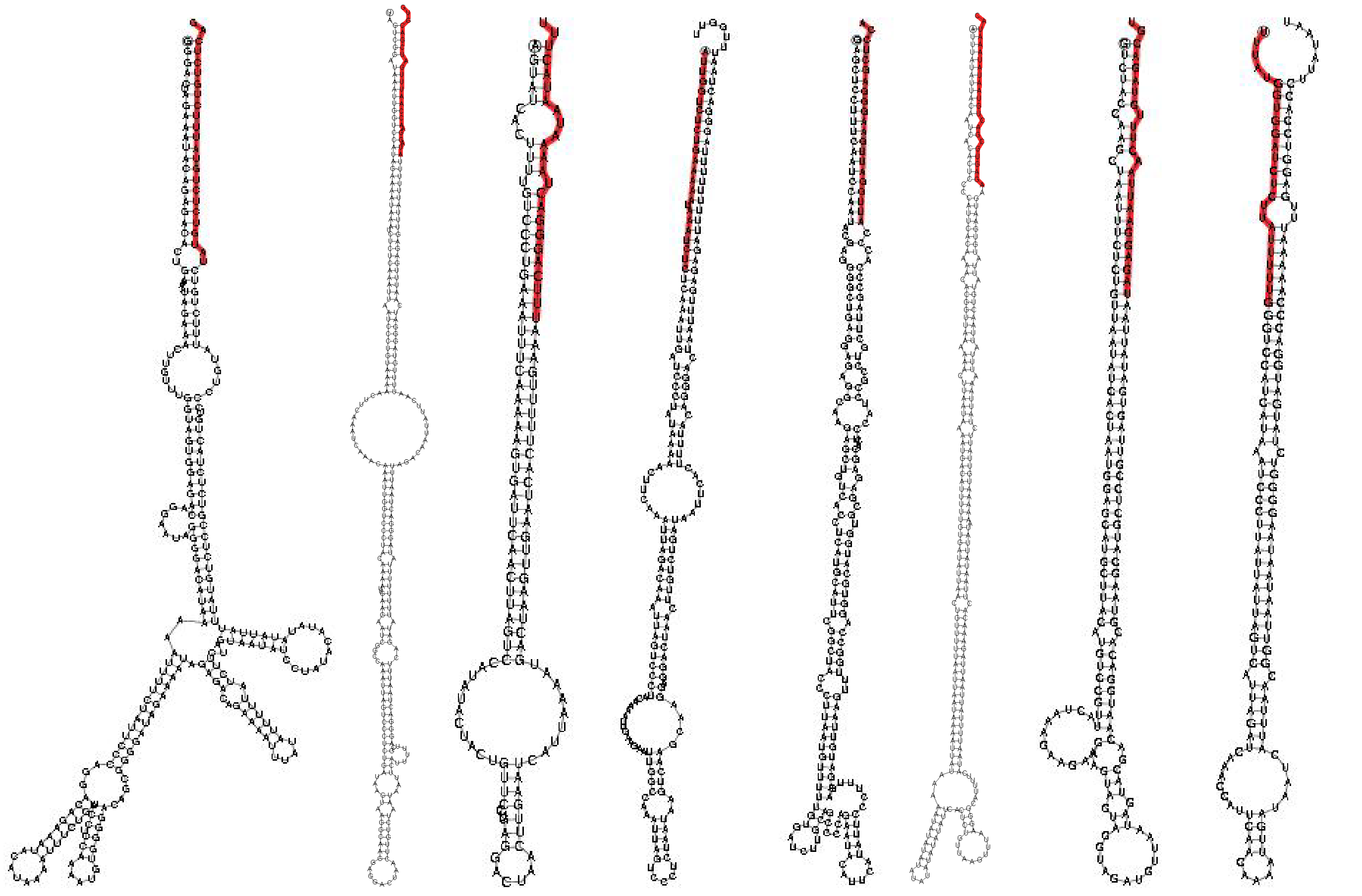

novel_90 novel_92 novel_93 novel_94 novel_95 novel_96 novel_97 novel_98

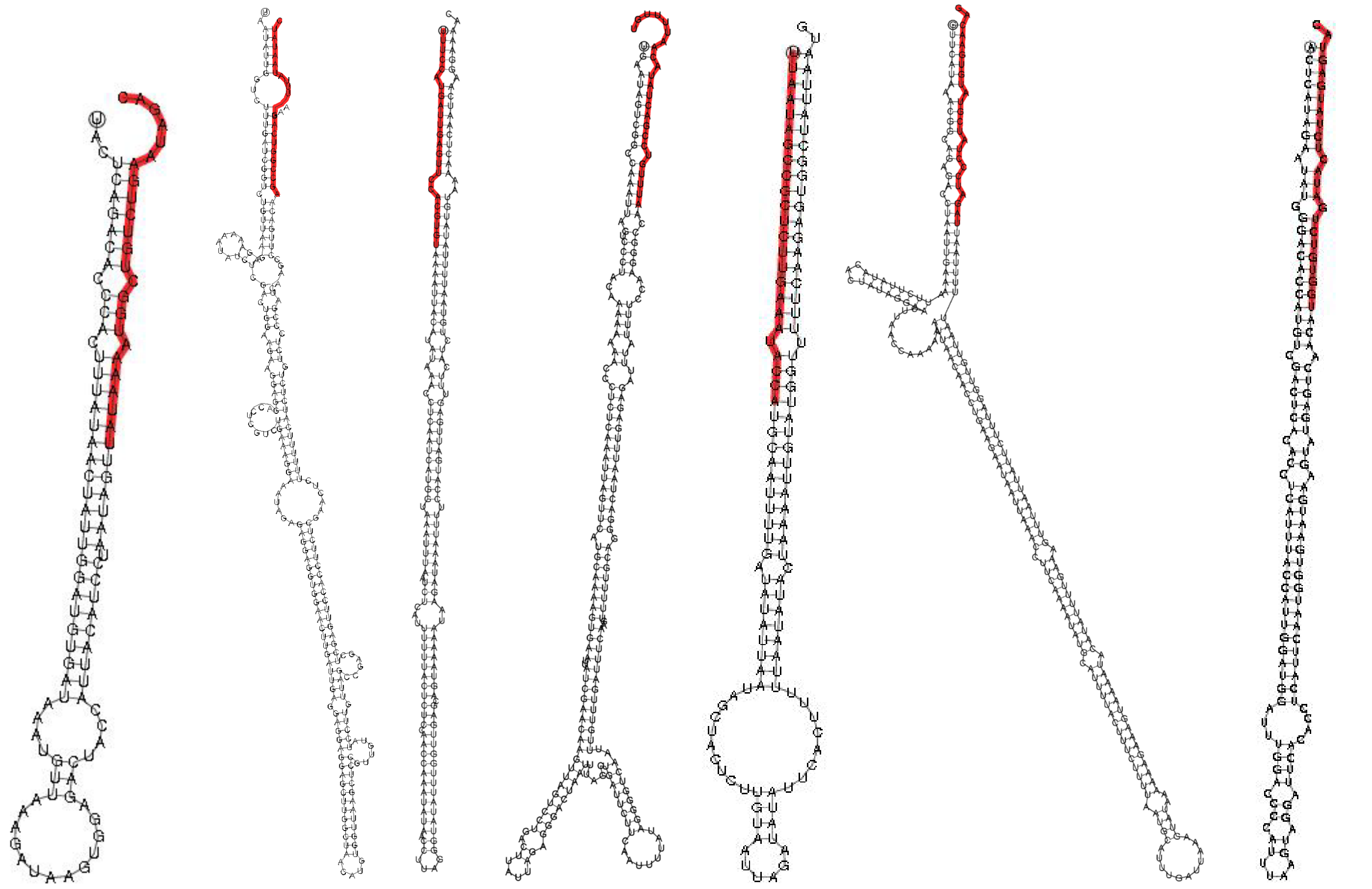

novel_99 **novel_100** **novel_101** **novel_102** **novel_103** **novel_105** **novel_107**

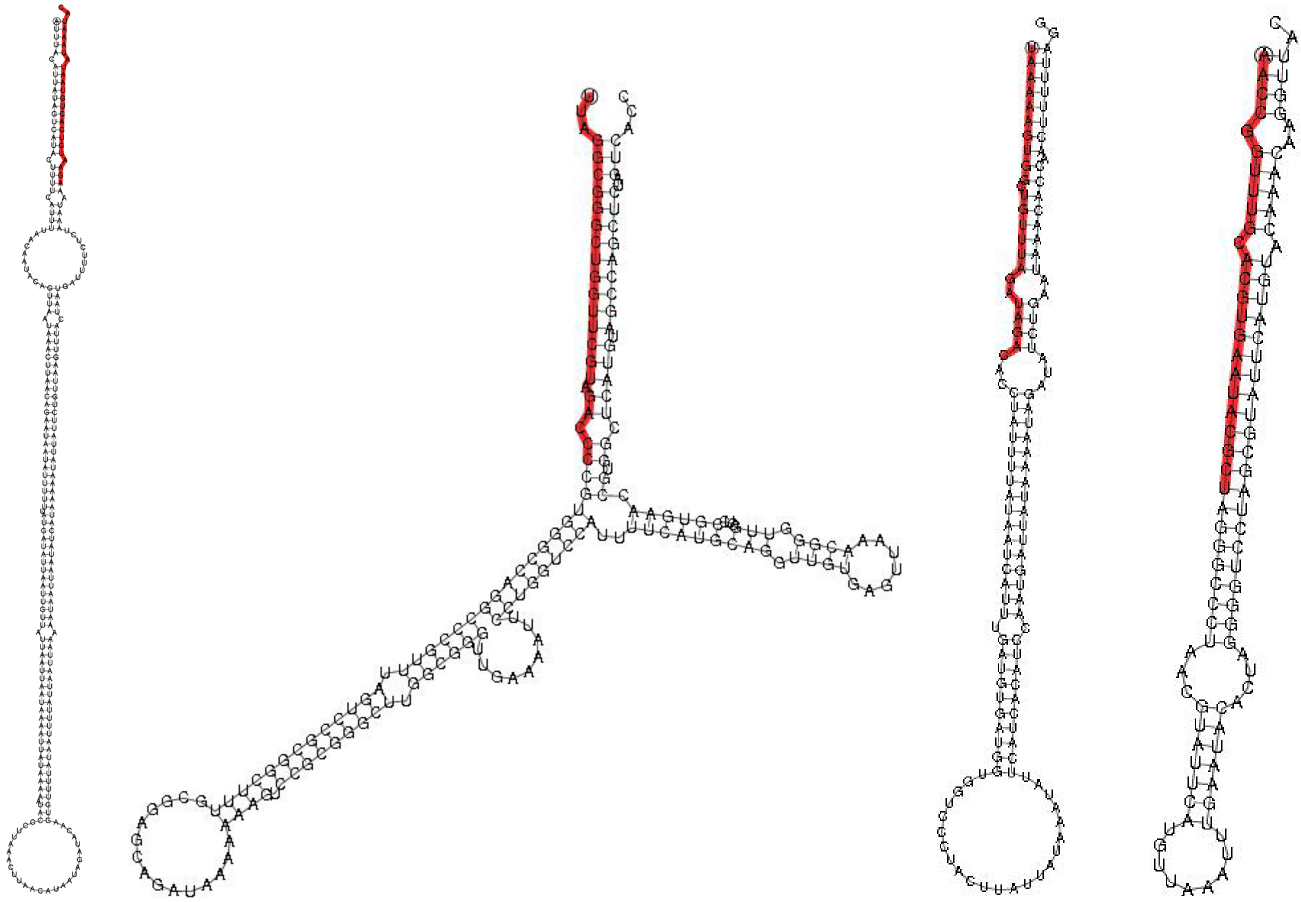

novel_110 **novel_111** **novel_113** **novel_114**

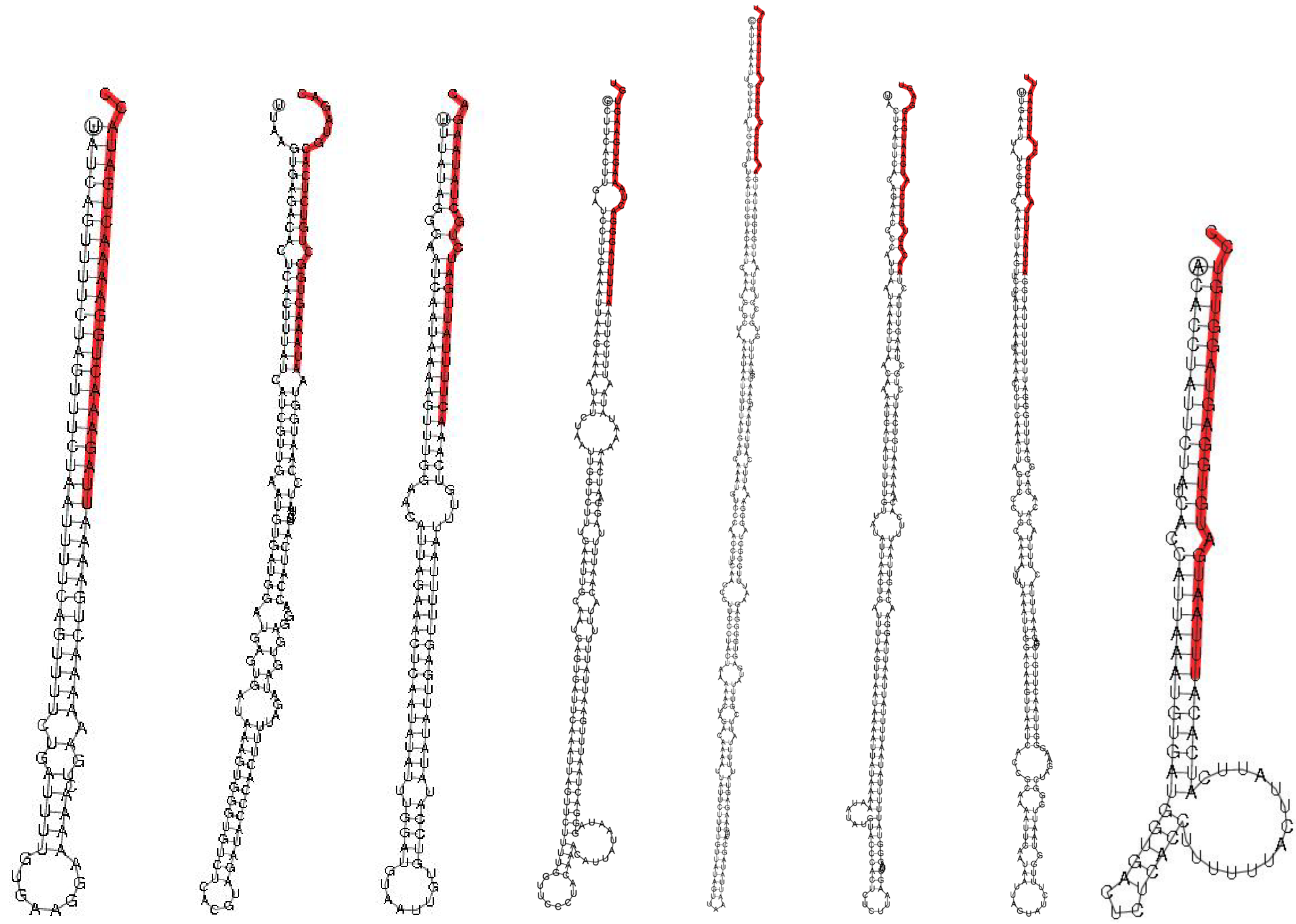

novel_115 **novel_116** **novel_117** **novel_118** **novel_119** **novel_120** **novel_121** **novel_122**

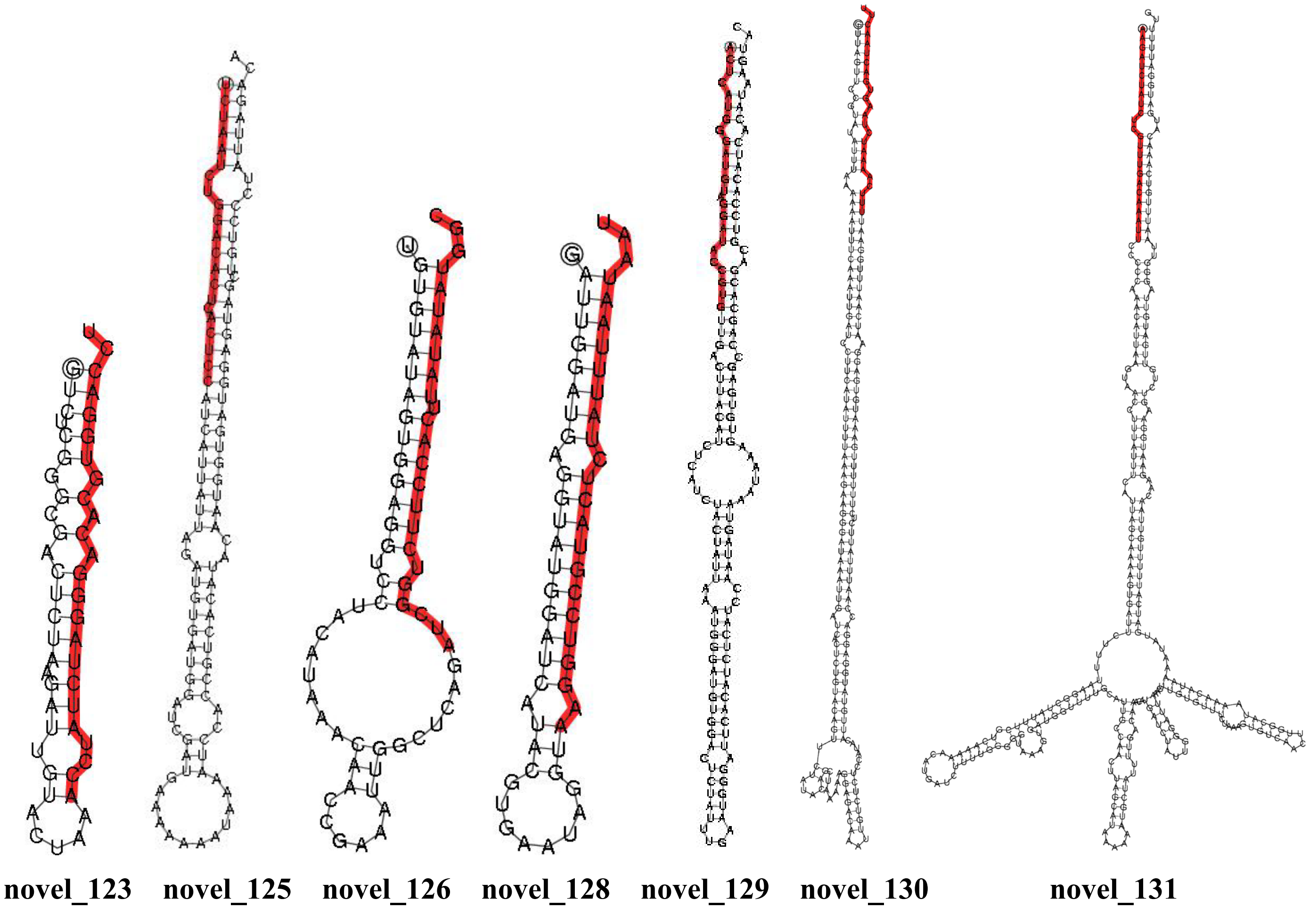

novel_123 **novel_125** **novel_126** **novel_128** **novel_129** **novel_130** **novel_131**

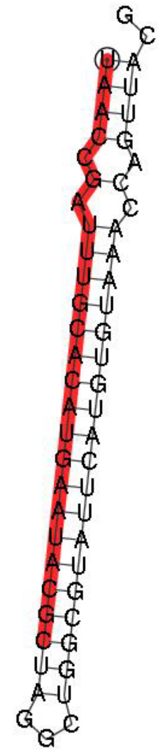

novel_132

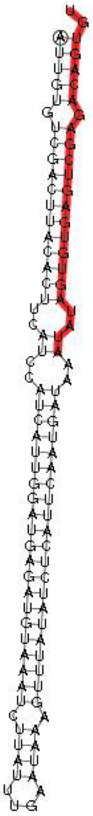

novel_133

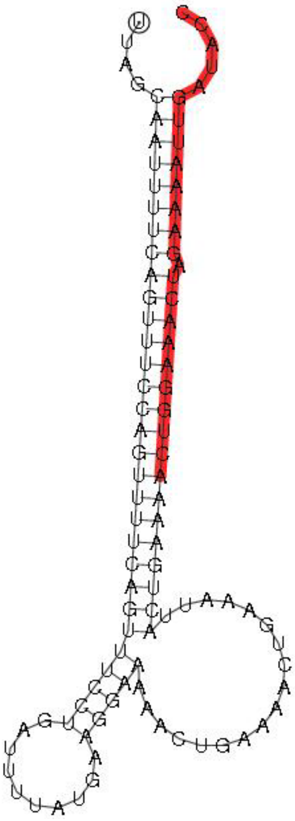

novel_134

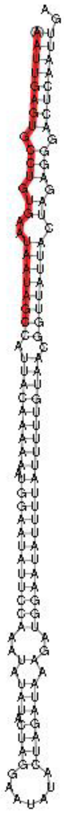

novel_136

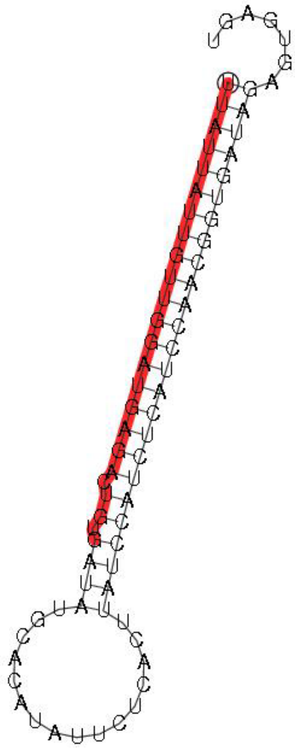

novel_138

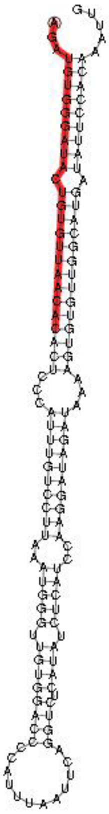

novel_139

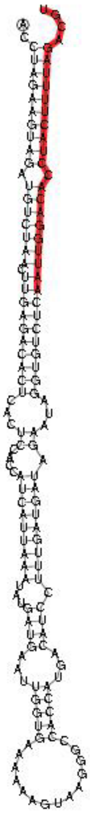

novel_140

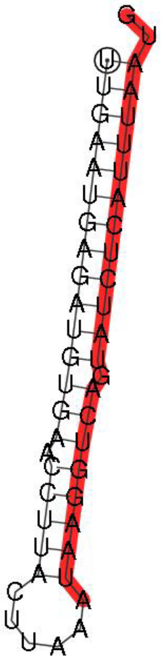

novel_144

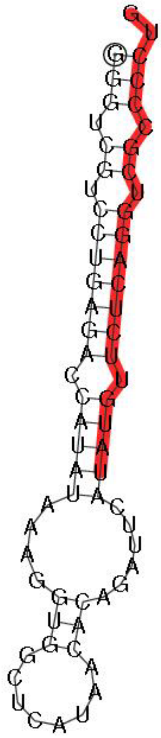

novel_145

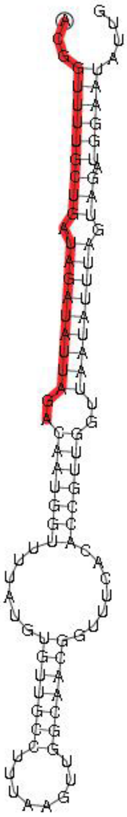

novel_146

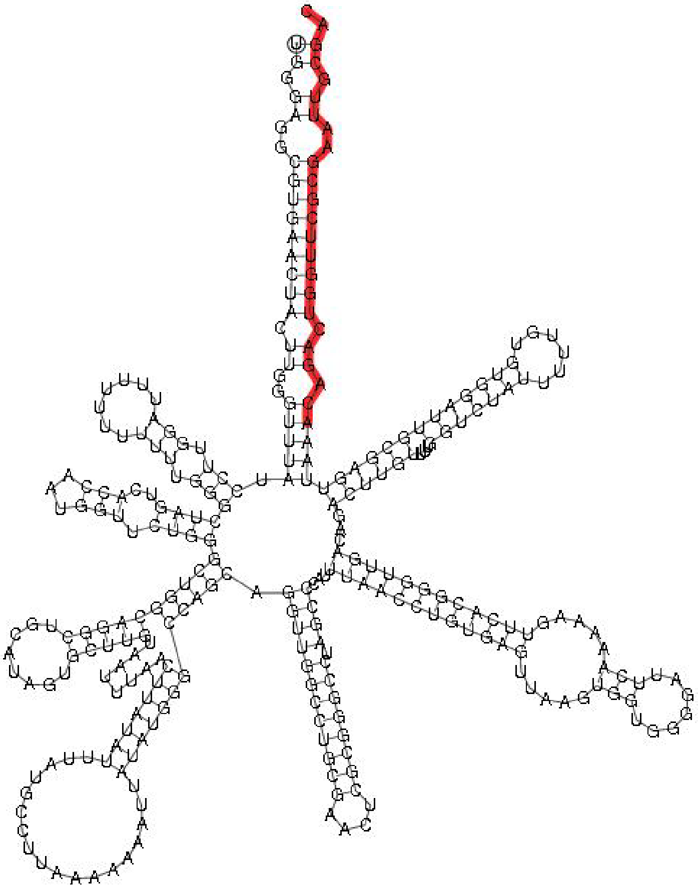

novel_147

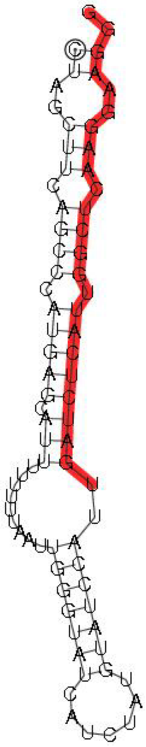

novel_149

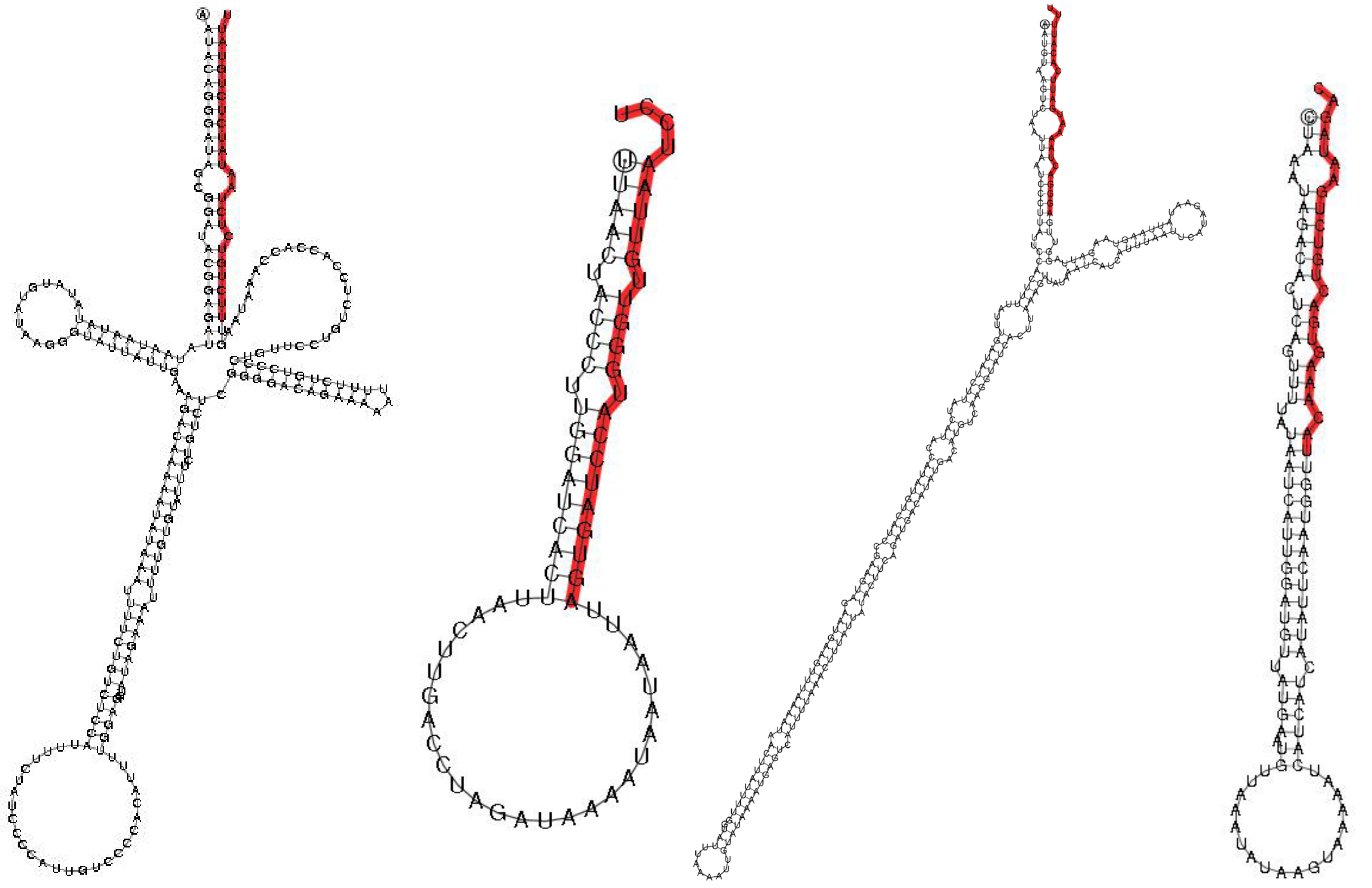

novel_148 novel_150 novel_151 novel_152

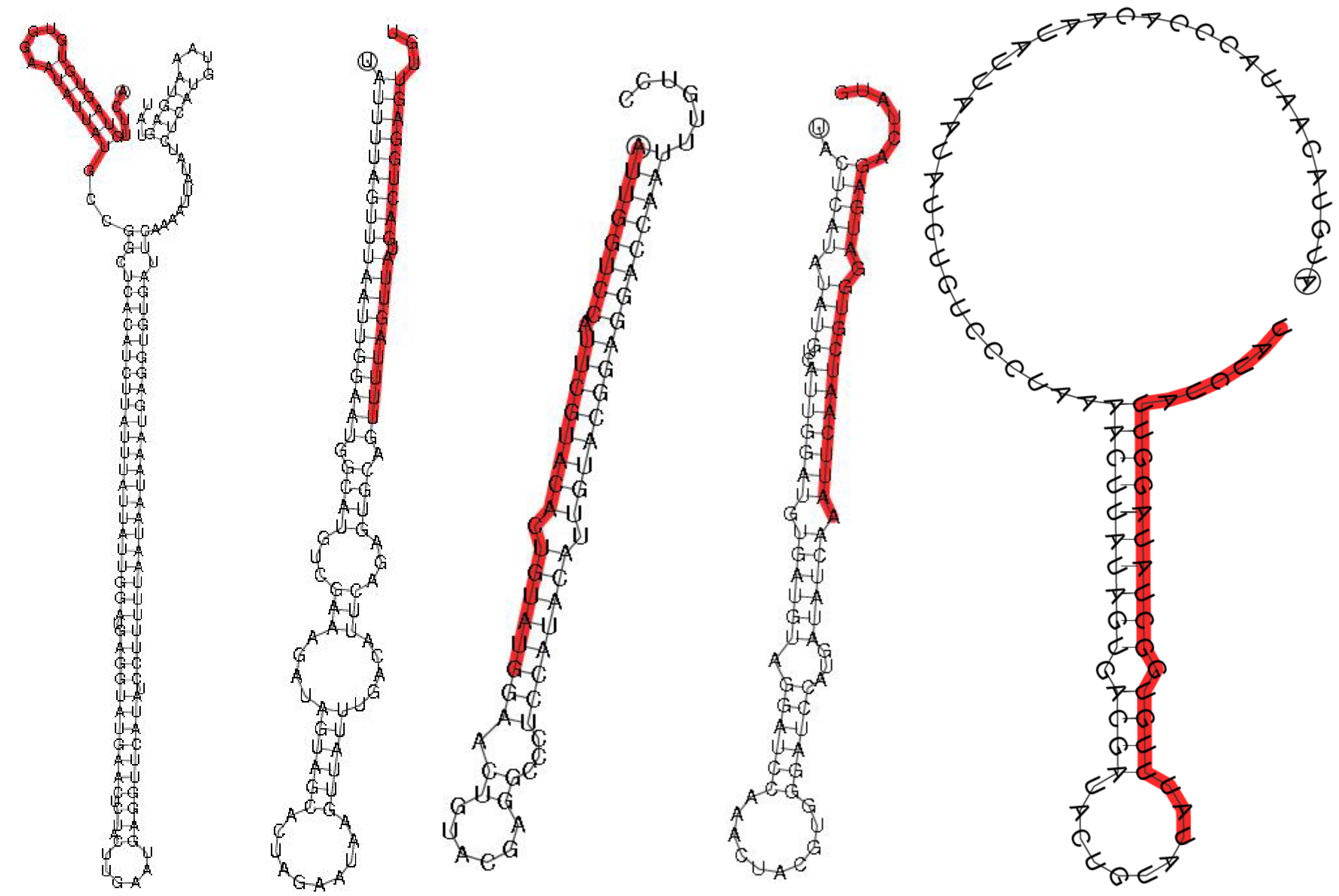

novel_154 novel_155 novel_156 novel_157 novel_158

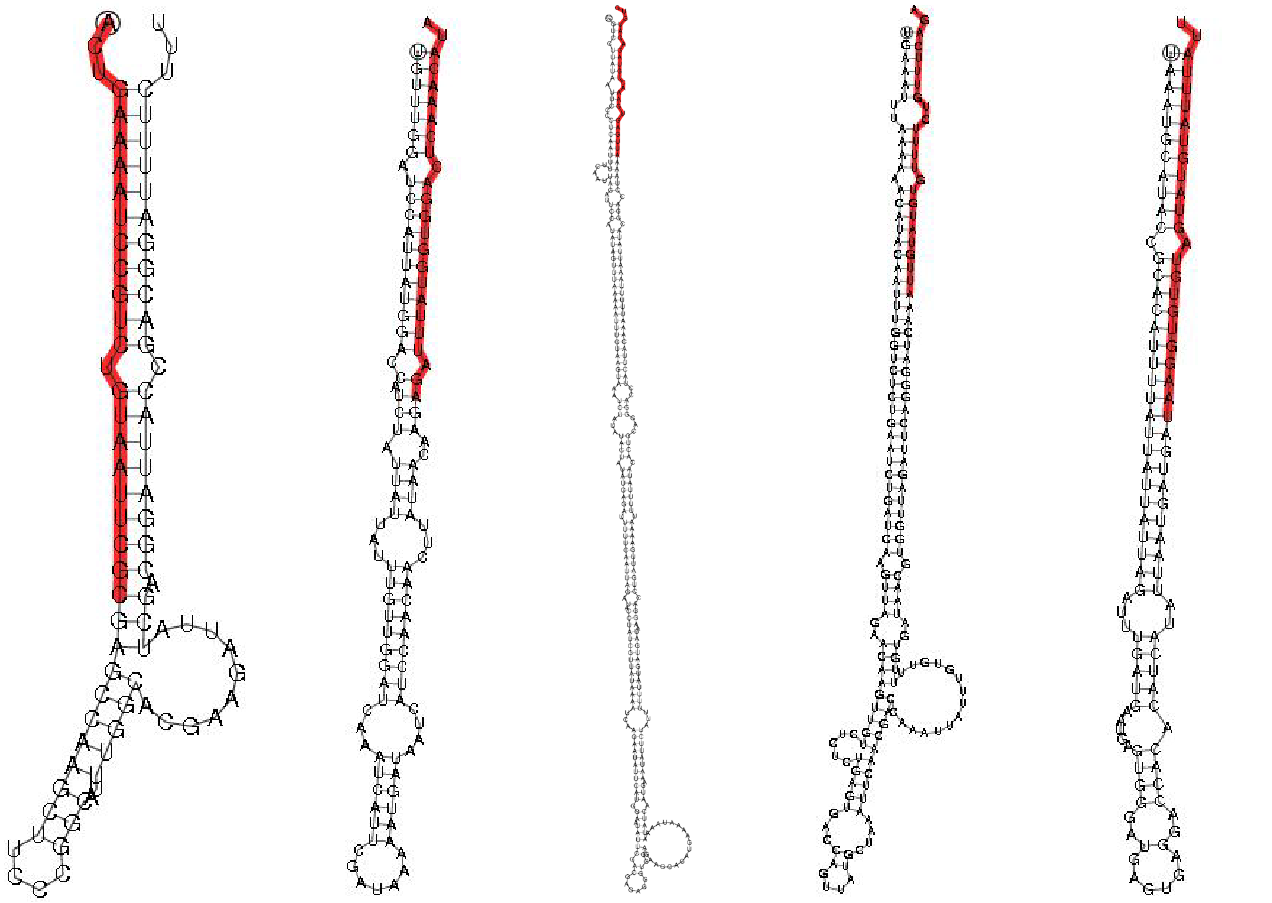

novel_159 **novel_161** **novel_162** **novel_163** **novel_164**

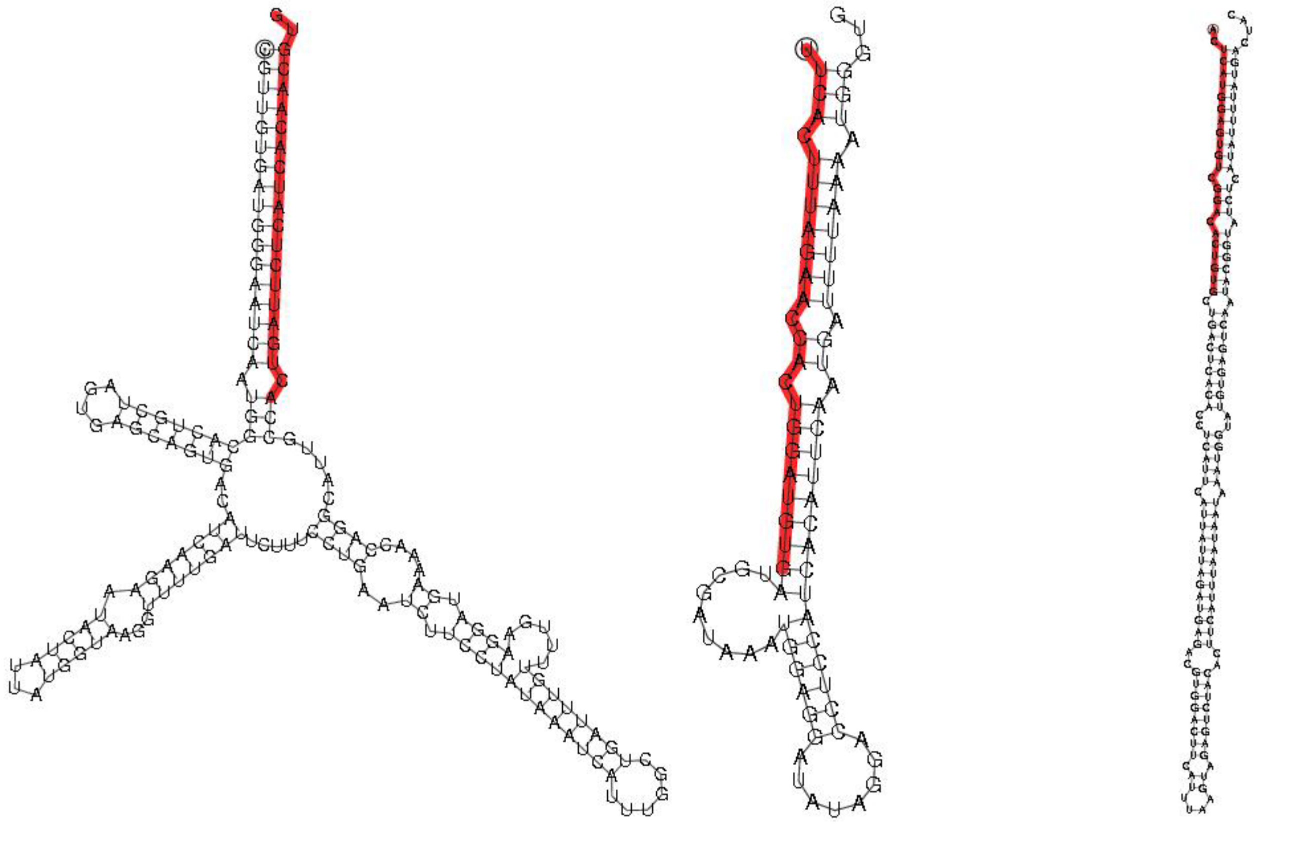

novel_165 **novel_166** **novel_170**

Supplementary Figure S4: The predicted secondary structures of novel miRNAs. The entire sequence is of the miRNA precursor, and the red highlight is the position of the mature miRNA.

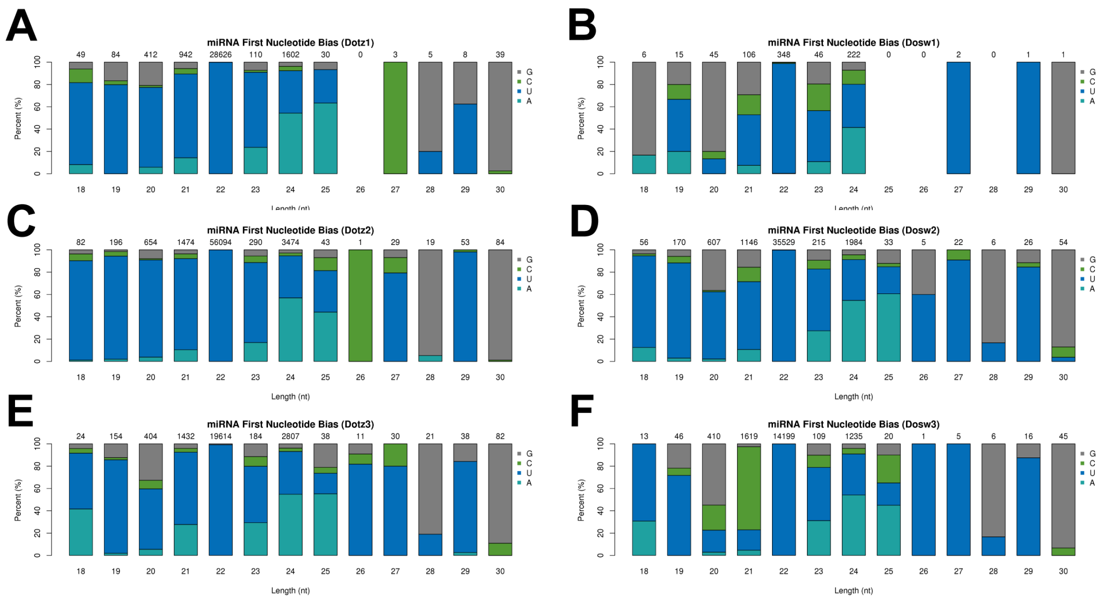

Supplementary Figure S5: First base type of the novel miRNA in six samples of 18-30 nt length. Dotz1 (A), Dosw1 (B), Dotz2 (C), Dosw2 (D), Dotz3 (E) and Dosw3 (F). The x-axis is the length of miRNAs, and the y-axis is the percentage of A/U/C/G at the first base of miRNA at that length.

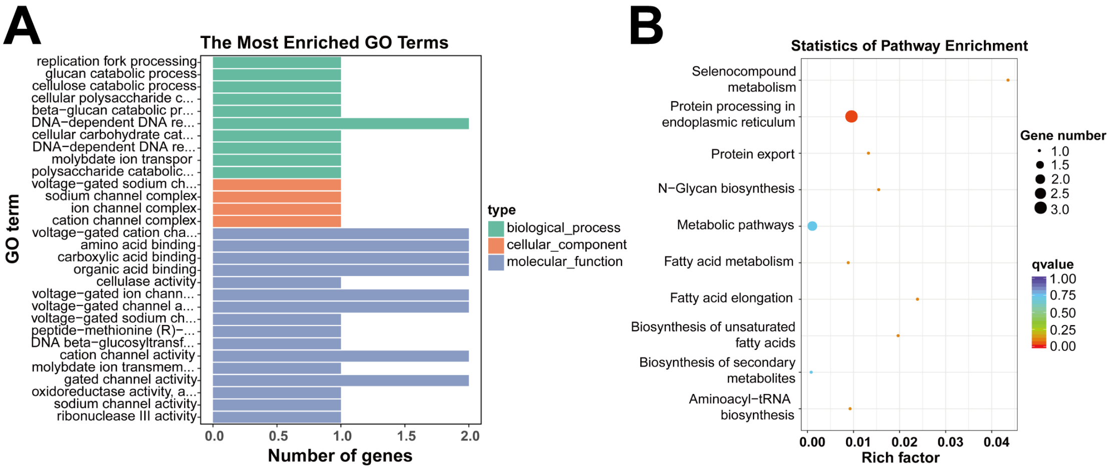

Supplementary Figure S6: Supplementary Figure S6: GO and KEGG analysis of the miRNAs in the co-expression network. (A) GO enrichment analysis of miRNA targets. The x-axis represents the number of genes, and the y-axis represents the GO term. (B) KEGG pathway enrichment analysis of miRNA. The x-axis represents the rich factor, whereas the y-axis represents the KEGG pathway term. The size of the dots represents the number of genes, and the color of the dots represents q-value.
